# Faster sorting algorithms discovered using deep reinforcement learning

**DOI:** 10.1038/s41586-023-06004-9

**Published:** 2023-06-07

**Authors:** Daniel J. Mankowitz, Andrea Michi, Anton Zhernov, Marco Gelmi, Marco Selvi, Cosmin Paduraru, Edouard Leurent, Shariq Iqbal, Jean-Baptiste Lespiau, Alex Ahern, Thomas Köppe, Kevin Millikin, Stephen Gaffney, Sophie Elster, Jackson Broshear, Chris Gamble, Kieran Milan, Robert Tung, Minjae Hwang, Taylan Cemgil, Mohammadamin Barekatain, Yujia Li, Amol Mandhane, Thomas Hubert, Julian Schrittwieser, Demis Hassabis, Pushmeet Kohli, Martin Riedmiller, Oriol Vinyals, David Silver

**Affiliations:** 1 Deepmind, London, UK; 2 Google, Mountain View, CA USA

**Keywords:** Computer science, Software

## Abstract

Fundamental algorithms such as sorting or hashing are used trillions of times on any given day^1^. As demand for computation grows, it has become critical for these algorithms to be as performant as possible. Whereas remarkable progress has been achieved in the past^2^, making further improvements on the efficiency of these routines has proved challenging for both human scientists and computational approaches. Here we show how artificial intelligence can go beyond the current state of the art by discovering hitherto unknown routines. To realize this, we formulated the task of finding a better sorting routine as a single-player game. We then trained a new deep reinforcement learning agent, AlphaDev, to play this game. AlphaDev discovered small sorting algorithms from scratch that outperformed previously known human benchmarks. These algorithms have been integrated into the LLVM standard C++ sort library^3^. This change to this part of the sort library represents the replacement of a component with an algorithm that has been automatically discovered using reinforcement learning. We also present results in extra domains, showcasing the generality of the approach.

## Abstract (web summary)

Artificial intelligence goes beyond the current state of the art by discovering unknown, faster sorting algorithms as a single-player game using a deep reinforcement learning agent. These algorithms are now used in the standard C++ sort library.

## Main

Human intuition and know-how have been crucial in improving algorithms. However, many algorithms have reached a stage whereby human experts have not been able to optimize them further, leading to an ever-growing computational bottleneck. The work in classical program synthesis literature, spanning many decades, aims to generate correct programs and/or optimize programs using proxies for latency. These include enumerative search techniques^4–7^ and stochastic search^5,6,8–10^ as well as the more recent trend of using deep learning in program synthesis for generating correct programs^11–16^. Using deep reinforcement learning (DRL), we can take this a step further by generating correct and performant algorithms by optimizing for actual measured latency at the CPU instruction level, by more efficiently searching and considering the space of correct and fast programs compared to previous work.

One of the fundamental questions in computer science is how to sort a sequence^17–20^. This is taught in elementary computer science classes around the world^21,22^ and is used ubiquitously by a vast range of applications^23–25^. Decades of computer science research have focused on discovering and optimizing sorting algorithms^26–28^. A key component of practical solutions is a small sort over a short sequence of elements; this algorithm is called repeatedly when sorting large arrays that use divide-and-conquer approaches^29^. In this work, we focus on two types of small sort algorithm: (1) the fixed sort and (2) the variable sort. Fixed sort algorithms sort sequences of a fixed length (for example, sort 3 can only sort sequences of length 3), whereas variable sort algorithms can sort a sequence of varying size (for example, variable sort 5 can sort sequences ranging from one to five elements).

We formulate the problem of discovering new, efficient sorting algorithms as a single-player game that we refer to as AssemblyGame. In this game, the player selects a series of low-level CPU instructions, which we refer to as assembly instructions^30^, to combine to yield a new and efficient sorting algorithm. This is challenging as the player needs to consider the combinatorial space of assembly instructions to yield an algorithm that is both provably correct and fast. The hardness of the AssemblyGame arises not only from the size of the search space, which is similar to extremely challenging games such as chess (10^120^ games)^31^ and Go (10^700^ games)^32^, but also from the nature of the reward function. A single incorrect instruction in the AssemblyGame can potentially invalidate the entire algorithm, making exploration in this space of games incredibly challenging.

To play the game, we introduce AlphaDev, a learning agent that is trained to search for correct and efficient algorithms. This agent is comprised of two core components, namely (1) a learning algorithm and (2) a representation function. The AlphaDev learning algorithm can incorporate both DRL as well as stochastic search optimization algorithms to play AssemblyGame. The primary learning algorithm in AlphaDev is an extension of AlphaZero^33^, a well-known DRL algorithm, in which a neural network is trained to guide a search to solve AssemblyGame. The representation function is interchangeable and captures the underlying structure of assembly programs. The primary AlphaDev representation is based on Transformers^34^.

Using AlphaDev, we have discovered fixed and variable sort algorithms from scratch that are both new and more efficient than the state-of-the-art human benchmarks. The fixed sort solutions for sort 3, sort 4 and sort 5 discovered by AlphaDev have been integrated into the standard sort function in the LLVM standard C++ library^3^. This library is used by several million users including universities and numerous international companies^35^. In addition, we analyse the new algorithm discoveries, compare AlphaDev to stochastic search optimization approaches and apply AlphaDev to further domains to showcase the generality of the approach.

## Representing algorithms as low-level CPU instructions

**Fig. 1:**
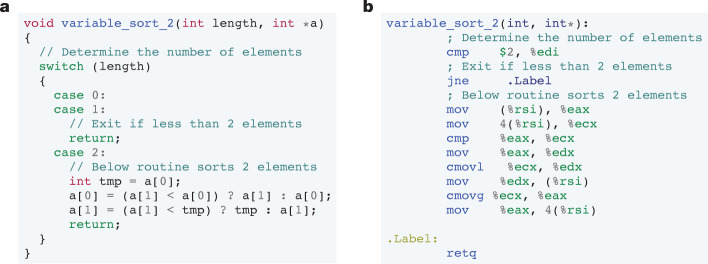
The relationship between C++ and assembly programs. **a**, A C++ implementation of a variable sort 2 function that sorts any input sequence of up to two elements. **b**, The C++ implementation in **a** is compiled to this equivalent low-level assembly representation.

When compiling algorithms to machine code from a high level language such as C++ (for example, the sorting function in Fig. 1a), the algorithm is first compiled into assembly (Fig. 1b). The assembler then converts the assembly program into executable machine code. In this work, we optimize algorithms at the assembly level^30^. In a typical assembly program, the values are copied from memory into registers, manipulated between registers and then written back to memory. The set of assembly instructions supported depends on the processor architecture. For the purposes of this work, we focus on a subset of assembly instructions supported by the x86 processor architecture using the AT&T syntax^36^. Each instruction is of the format Opcode⟨Operand_A_, Operand_B_⟩. An example instruction is mov<A,B>, which is defined as move a value from source (A) to destination (B). Further instruction definitions such as compare (cmp<A,B>), conditional move (cmovX<A,B>) and jump (jX<A>) can be found in Extended Data Table 1. In the example in Fig. 1b, %eax, %ecx, %edx, %edi correspond to four different register locations and (%rsi), 4(%rsi) correspond to two different memory locations. The symbol $2 is a placeholder for a constant value, which corresponds to the length of the vector in this example. We use the terms assembly program and assembly algorithm interchangeably in this work. This is because AlphaDev builds an assembly program from scratch, from an initially unordered set of instructions, each time it plays AssemblyGame, defining a new and efficient algorithm.

## DRL for discovering faster algorithms

**Fig. 2:**
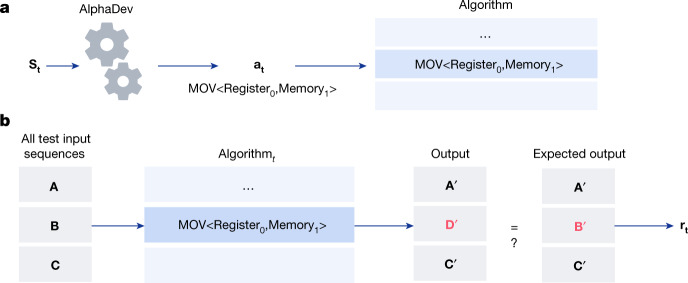
The AssemblyGame and algorithm correctness computation. **a**, The AssemblyGame is played by AlphaDev, which receives as input the current assembly algorithm generated thus far **S**_**t**_ and plays the game by selecting an action to execute. In this example, the action is a mov<Register_0_,Memory_1_> assembly instruction, which is appended to the current algorithm. The agent receives a reward that is a function of the algorithm’s correctness, discussed in **b**, as well as the algorithm’s latency. The game is won by the player discovering a low latency, correct algorithm. **b**, The program correctness and latency computations are used to compute the reward *r*_t_. In this example, test sequences are input to the algorithm; for example, in the case of sorting three elements, test inputs comprise all sequences of unsorted elements of length 3. For each sequence, the algorithm output is compared to the expected output (in the case of sorting, the expected output is the sorted elements). In this example, the output ${\bf{D}}{\boldsymbol{{\prime} }}$ does not match the expected output ${\bf{B}}{\boldsymbol{{\prime} }}$ and the algorithm is therefore incorrect.

In this section, we formulate optimizing algorithms at the CPU instruction level as a reinforcement learning (RL) problem^37^, in which the environment is modelled as a single-player game that we refer to as AssemblyGame. Each state in this game is defined as a vector **S**_**t**_ = ⟨**P**_**t**_, **Z**_**t**_⟩ where **P**_**t**_ is a representation of the algorithm generated thus far in the game and **Z**_**t**_ represents the state of memory and registers after executing the current algorithm on a set of predefined inputs. As seen in Fig. 2a, at timestep *t*, the player receives the current state **S**_**t**_ and executes an action **a**_**t**_. This involves appending a legal assembly instruction (for example, mov<A,B>) to the current algorithm generated thus far. A reward *r*_**t**_is received that comprises both a measure of algorithm correctness and latency. Algorithm correctness (Fig. 2b) involves inputting a set of *N* test sequences into the current algorithm **P**_**t**_ to generate *N* outputs. These outputs are then compared to the expected outputs and a correctness reward *r*_**t**_ is computed. Latency rewards can be generated by either (1) penalizing the agent for increasing the length of the algorithm (when length and latency are highly correlated) that we refer to as the algorithm length reward, or (2) measuring the actual latency of the algorithm. The game is executed for a limited number of steps, after which the game is terminated. Winning the game corresponds to generating a correct, low-latency algorithm using assembly instructions. Losing the game corresponds to generating an incorrect algorithm or a correct but inefficient algorithm.

We refer to the agent that plays this single-player game as AlphaDev. The agent’s primary learning algorithm is an extension of the AlphaZero agent^32^ and guides a Monte Carlo tree search (MCTS) planning procedure using a deep neural network^33,38^. The input to the neural network is the state **S**_**t**_ and the output is a policy and value prediction. The policy prediction is a distribution over actions and the value function is a prediction of the cumulative returns *R* that the agent should expect to receive from the current state **S**_**t**_. During a game, the agent receives as input the current state **S**_**t**_. The agent then executes an MCTS procedure and uses this to select the next action to take. The generated games are then used to update the network’s parameters, enabling the agent to learn.

**Extended Data Fig. 1:**
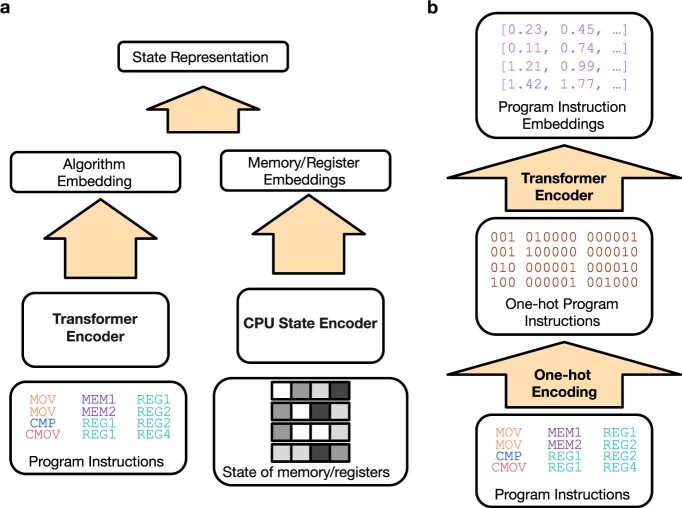
The AlphaDev representation network architecture. **(a)** The AlphaDev representation network comprises a Transformer Encoder network that receives as input the assembly algorithm generated thus far. It also contains a CPU State Encoder network that receives as input the current state of memory and registers. The exact architecture and hyperparameters can be found in the Supplementary Information, Appendix A. **(b)** Before inputting instructions into the Transformer Encoder network, each program instruction’s opcode and operands are converted to one-hot encodings and concatenated. The resulting encoding is then fed into the Transformer Encoder network.

It is critical that AlphaDev has a representation^39,40^ capable of representing complex algorithmic structures to efficiently explore the space of instructions. To achieve this, we introduce the AlphaDev representation network (Extended Data Fig. 1a). This network comprises two components, namely (1) a transformer encoder network that provides the agent with a representation of the algorithm structure, and (2) the CPU state encoder network that helps the agent predict how the algorithm affects the dynamics of memory and registers. The CPU state encoder network comprises a multilayer perceptron that receives as input the state of each register and memory location for a given set of inputs. These networks each output embeddings that are combined to yield the AlphaDev state representation.

### Transformer encoder

Transformers are natural text encoders and have had much success with language models recently^14,34,41^. As such, this motivated us to adapt the standard transformer to model assembly instructions. We developed and incorporated a transformer encoder, our adaptation of the MultiQuery transformer encoder^42^, into the AlphaDev representation network to represent the assembly instructions. Each assembly instruction’s Opcode and corresponding Operands are converted to one-hot encodings and concatenated to form the raw input sequence. This is fed through a multilayer transformer encoder, which maps it to corresponding embedding vectors (see Extended Data Fig. 1b for an illustration).

### Latency value functions

Latency is an important reward signal that is used to guide the agent in discovering performant algorithms. To better estimate latency, we implemented a dual value function setup, whereby AlphaDev has two value function heads: one predicting algorithm correctness and the second predicting algorithm latency. The latency head is used to directly predict the latency of a given program by using the program’s actual computed latency as a Monte Carlo target for AlphaDev during training. This dual-head approach achieved substantially better results than the vanilla, single head value function setup when optimizing for real latency.

## Results

### Discovering faster sort algorithms

We trained the AlphaDev agent from scratch to generate a range of fixed sort and variable sort algorithms that are both correct and achieve lower latency than the state-of-the-art human benchmarks.

### Fixed sorting algorithms

We considered three fundamental algorithms: sort 3, sort 4 and sort 5. The state-of-the-art human benchmarks for these algorithms are sorting networks^43^ as they generate efficient, conditional branchless assembly code. This means that all instructions are executed sequentially and there is no branching involved. Improving on these algorithms is challenging as they are already highly optimized. As seen in Table 1a, AlphaDev is able to find algorithms with fewer instructions than the human benchmarks for sort 3 and sort 5 and matches the state-of-the-art performance on sort 4. These shorter algorithms do indeed lead to lower latency as the algorithm length and latency are correlated for the conditional branchless case; see Appendix B in Supplementary Information for more details. We also explored scaling to slightly larger sorts using a variant of AlphaDev. We managed to save three instructions on sort 6, two instructions on sort 7 and one instruction on sort 8, which provides a promising basis for future work. See Appendix C in Supplementary Information for an overview of the approach.

**Table 1:** AlphaDev performance when optimizing for algorithm length and latency

(a) Algorithm	AlphaDev	Human benchmarks
	Length	Length
Sort 3	17	18
Sort 4	28	28
Sort 5	42	46
VarSort3	21	33
VarSort4	37	66
VarSort5	63	115
VarInt	27	31

(b) Algorithm	AlphaDev	Human benchmarks
	Latency ± (lower, upper)	Latency ± (lower, upper)
VarSort3	236,498 ± (235,898, 236,887)	246,040 ± (245,331, 246,470)
VarSort4	279,339 ± (278,791, 279,851)	294,963 ± (294,514, 295,618)
VarSort5	312,079 ± (311,515, 312,787)	331,198 ± (330,717, 331,850)
VarInt	97,184 ± (96,885, 97,847)	295,358 ± (293,923, 296,297)
Competitive	75,973 ± (75,420, 76,638)	86,056 ± (85,630, 86,913)

**a**, AlphaDev performance, compared to the human benchmarks, when optimizing for algorithm length. AlphaDev discovers algorithms from scratch that match or improve on the human benchmarks in each case. **b**, AlphaDev performance, compared to the human benchmarks, when optimizing directly for latency. In this setup, AlphaDev discovers algorithms that have significantly lower latency than the human benchmarks in each case. The confidence intervals are represented as latency ± (lower, upper), in which latency corresponds to the fifth percentile of latency measurements across 100 different machines. Lower and upper refer to the bounds of the 95% confidence interval for this percentile.

### Variable sorting algorithms

We considered three variable sorting algorithms: VarSort3, VarSort4 and VarSort5. The human benchmark in each case is defined as an algorithm that, for a given input length, calls the corresponding sorting network. In this case, branching is required, which greatly increases the complexity of the problem as the agent needs to (1) determine how many subalgorithms it needs to construct and (2) build the body of the main algorithm in parallel. The agent may also need to call subalgorithms from other subalgorithms. In this case, optimizing for length leads to significantly shorter algorithms compared to the human benchmarks as seen in Table 1a. However, owing to the complexities introduced by branching, latency and length are not always correlated; see Supplementary Information for more details. As such, we implemented a procedure that measures the actual latency of the programs by taking the fifth percentile of latency measurements across 100 different machines, with computed confidence intervals^44^, and optimize this metric. See Methods for the full benchmarking setup. When optimizing for latency, the agent improves significantly on the human benchmarks in each case as seen in Table 1b.

### New algorithm discoveries

The solutions discovered by AlphaDev include new and exciting algorithmic discoveries that lead to more efficient performance. In the fixed sort setting, we found that AlphaDev discovered two interesting sequences of instructions that, when applied to a sorting network algorithm, reduce the algorithm by one assembly instruction each time. We refer to each sequence of instructions as (1) the AlphaDev swap move and (2) the AlphaDev copy move respectively.

### AlphaDev swap move

**Fig. 3:**
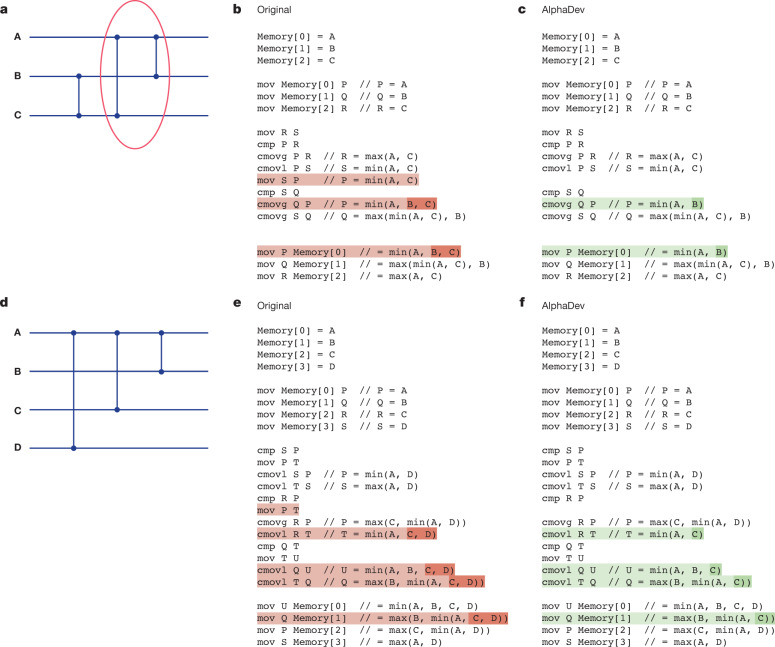
Sorting networks and algorithmic improvements discovered by AlphaDev. **a**, An optimal classic sorting network for three inputs. The circled comparators have been improved by AlphaDev. See the AlphaDev swap move for more details. **b**,**c**, The assembly pseudocode before applying the AlphaDev swap move (**b**) and after applying the AlphaDev swap move (**c**), resulting in the removal of a single instruction. **d**, An optimal classic sorting network comparator configuration that has been improved by AlphaDev. See the AlphaDev copy move for more details. **e**,**f**, The assembly pseudocode before applying the AlphaDev copy move (**e**) and after applying the AlphaDev copy move (**f**), resulting in the removal of a single instruction.

Figure 3a presents an optimal sorting network for three elements (see Methods for an overview of sorting networks). We will explain how AlphaDev has improved the circled network segment. There are many variants of this structure that are found in sorting networks of various sizes, and the same argument applies in each case. The circled part of the network (last two comparators) can be seen as a sequence of instructions that takes an input sequence ⟨A, B, C⟩ and transforms each input as shown in Table 2a (left). However, a comparator on wires B and C precedes this operator and therefore input sequences where B ≤ C are guaranteed. This means that it is enough to compute min(A, B) as the first output instead of min(A, B, C) as shown in Table 2a (right). The pseudocode difference between Fig. 3b,c demonstrates how the AlphaDev swap move saves one instruction each time it is applied.

**Table 2:** Analysis of the AlphaDev swap and copy moves

(a) Input	Original output	AlphaDev swap move
A	min(A, B, C)	**min(A, B)**
B	max(min(A, C), B)	max(min(A, C), B)
C	max(A, C)	max(A, C)

(b) Input	Original output	AlphaDev copy move
A	min(A, B, C, D)	min(A, B, C, D)
B	max(B, min(A, C, D))	**max(B, min(A, C))**
C	max(C, min(A, D))	max(C, min(A, D))
D	max(A, D)	max(A, D)

**a**, Left shows the transformation applied to inputs A, B and C in a classic sorting network when applying the circled operator in Fig. 3a. Right shows the AlphaDev swap move transformation applied in place of the circled operator. Note the new transformation in bold that saves a single instruction each time it is applied. **b**, Left shows the transformation applied to inputs A, B, C and D according to the sorting network configuration in Fig. 3d. Right shows the AlphaDev copy move transformation applied to this sorting network configuration. The transformation in bold indicates the change made by the copy move, saving an instruction each time it is applied.

### AlphaDev copy move

Figure 3d presents a sorting network configuration, consisting of three comparators, that is applied across four wires. This configuration is found in a sort 8 sorting network and corresponds to an operator taking four inputs ⟨A, B, C, D⟩ and transforming them into four outputs as seen in Table 2b (on the left). One can show that as part of sort 8, the input that flows into the operator satisfies the following inequality: ${\rm{D}}\ge \min ({\rm{A}},{\rm{C}})$. This means that the operator can be improved by applying the AlphaDev copy move that is defined in Table 2b (on the right), resulting in one instruction less than the original operator. The code difference between the original operator and the code after applying the AlphaDev copy move is visualized in Fig. 3e,f, respectively.

### New variable sort algorithms

**Fig. 4:**
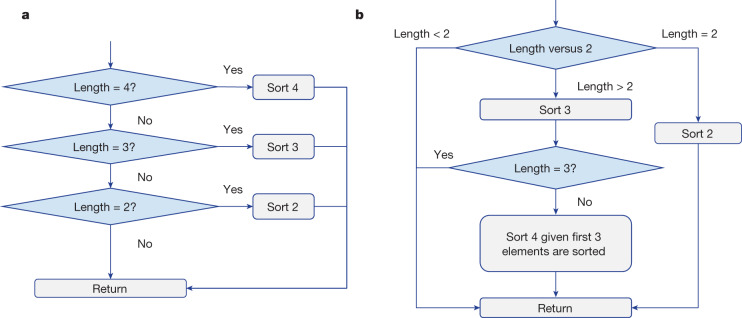
Fundamentally different algorithms discovered by AlphaDev. **a**, A flow diagram of the variable sort 4 (VarSort4) human benchmark algorithm. In this algorithm, a sequence of unsorted numbers are input into the algorithm. If the sequence length is four, three or two numbers, then the corresponding sort 4, sort 3 or sort 2 sorting network is called that sorts the resulting sequence. The result is then returned and output by the function. **b**, The VarSort4 algorithm discovered by AlphaDev. This algorithm also receives sequences of length four, three or two numbers as input. In this case, if the length is two, then it calls the sort 2 sorting network and returns. If the length is three then it calls sort 3 to sort the first three numbers and returns. If, however, the length is greater than three, then it calls sort 3, followed by a simplified sort 4 routine that sorts the remaining unsorted number. It is this part of the routine that results in significant latency savings.

The VarSort4 algorithm discovered by AlphaDev is particularly interesting. The flow diagram for the human benchmark algorithm and AlphaDev can be seen in Fig. 4a,b, respectively. The human benchmark algorithm determines the length of the input vector, and then calls the corresponding sorting network to sort the elements. The AlphaDev solution has a completely different approach as seen in Fig. 4b. If the length of the input vector is strictly greater than 2, then sort 3 is immediately called, resulting in the first three elements being sorted. If the vector is greater than three elements, then a simplified sort 4 algorithm is called that sorts the remaining unsorted elements in the input vector. It is this simplified part of the routine that yields significant gains in terms of algorithmic length and latency.

### Stochastic search optimization approaches

It is important to understand the advantages and limitations of RL compared to other approaches for program optimization. As such, we implemented a state-of-the-art stochastic superoptimization approach^8^, adapted it to the sort setting and used it as the learning algorithm in AlphaDev. We refer to this variant as AlphaDev-S (see Methods for more details). We run this algorithm with at least the same amount of resources and wall-clock time as AlphaDev. AlphaDev-S requires a prohibitive amount of time to optimize directly for latency as latency needs to be computed after every mutation. As such, AlphaDev-S optimizes for a latency proxy, namely algorithm length and, then, at the end of training, we search through all correct programs generated by AlphaDev-S and benchmark each one to find the lowest latency solution. In general, we find that AlphaDev consistently outperforms AlphaDev-S when learning from scratch without previous knowledge. In addition, as the size of the program increases, AlphaDev explores orders of magnitude fewer programs (12 million programs in the worst case) compared to AlphaDev-S (31 trillion programs in the worst case). This may be because AlphaDev is able to better explore the space of algorithms compared to the breadth-first stochastic search procedure that gets stuck more easily into local optima; see Methods for an overview of this exploration hypothesis. In addition, AlphaDev never evaluates latency during search as it uses the latency value function predictions and, because of this, only needs to compute actual measured latency on less than 0.002% of generated programs. When incorporating previous knowledge into AlphaDev-S, such as warm starting the learning algorithm with a near-optimal solution, AlphaDev-S is more computationally efficient for sort 3, sort 4 and sort 5 (branchless assembly algorithms) and also generates competitive low-latency algorithms to that of AlphaDev in each case. However, for algorithms that require branching (if–else statements), in which algorithm length and latency are not well correlated, AlphaDev discovers lower latency solutions than AlphaDev-S, even when warm starting this algorithm with a near-optimal solution. See Methods for an in-depth analysis of these algorithms.

### Generalization to additional domains

To test the generality of AlphaDev, we train the agent on a set of additional domains. These include a protocol buffer deserialization subroutine called VarInt, presented below, and a competitive coding problem (see Appendix D in Supplementary Information for more details). The competitive coding domain latency performance is reported in Table 1b.

Protocol Buffer is Google’s open-source data format used to serialize structured data^45^. This format is commonly used in cases in which performance or network load is of primary concern. The VarInt algorithm^46^ is a key component in both the serialization and deserialization processes. We trained the AlphaDev agent as in variable sort to optimize the VarInt deserialization function with respect to correctness and measured latency. For correctness, we reward the agent for correctly deserializing each input. We use a set of 80 inputs and corresponding outputs that cover common protobuf use cases. AlphaDev learns an optimized VarInt deserialization function and manages to significantly outperform the human benchmark for single valued inputs. Our agent discovers a branchless solution that is both shorter (Table 1a) and roughly three times faster than the human benchmark (Table 1b). In doing so, the agent also discovered a new VarInt assignment move in which AlphaDev learns to combine two operations into a single instruction leading to latency savings. See Appendix D.1 in Supplementary Information for a full overview of this move. This is a strong indication that AlphaDev is capable of generalizing to optimize non-trivial, real-world algorithms.

### Libc++ sort patch

The sort 3, sort 4 and sort 5 algorithms in the LLVM libc++ standard sorting library are called many times by larger sorting algorithms and are therefore fundamental components of the library. We reverse engineered the low-level assembly sorting algorithms discovered by AlphaDev for sort 3, sort 4 and sort 5 to C++ and discovered that our sort implementations led to improvements of up to 70% for sequences of a length of five and roughly 1.7% for sequences exceeding 250,000 elements. These improvements are for the uint32, uint64 and float data types for ARMv8, Intel Skylake and AMD Zen 2 CPU architectures; see Appendix E in Supplementary Information for the full performance tables. The performance improvements are due to both the branchless conditional assembly generated by AlphaDev as well as the new AlphaDev swap move. For sort 5, we used a 43 length algorithm discovered by AlphaDev, as it led to a more efficient C++ implementation. These algorithms were sent for review and have officially been included in the libc++ standard sorting library^3^. It is the first change to these sub-routines in over a decade. This is also the first time that any component in this sort library has been replaced by an algorithm that has been automatically discovered using reinforcement learning. We estimate that these routines are being called trillions of times every day^1,35,47^.

## Discussion

AlphaDev discovers new, state-of-the-art sorting algorithms from scratch that have been incorporated into the LLVM C++ library, used by millions of developers and applications around the world^23–25^. Both AlphaDev and stochastic search are powerful algorithms. An interesting direction for future research is to investigate combining these algorithms together to realize the complementary advantages of both approaches.

It is important to note that AlphaDev can, in theory, generalize to functions that do not require exhaustive verification of test cases. For example, hashing functions^48^ as well as cryptographic hashing functions^49^ define function correctness by the number of hashing collisions. Therefore, in this case, AlphaDev can optimize for minimizing collisions as well as latency. AlphaDev can also, in theory, optimize complicated logic components within the body of large, impressive functions. We hope that AlphaDev can provide interesting insights and inspire new approaches in both the artificial intelligence and program synthesis communities.

## Methods

### Background

#### AlphaZero

AlphaZero^33^ is an RL algorithm that leverages MCTS as a policy improvement operator. It consists of (1) a representation network *f*^rep^ that outputs a latent representation **h**_*t*_ of the state **S**_*t*_; and (2) a prediction network *f*^pred^ that predicts the expected return (the value) ${\hat{v}}_{t}$ and a policy (that is, distribution over the action space) ${\hat{\pi }}_{t}$ from a given latent state. The algorithm uses the true dynamics and reward when planning. MuZero^38^ is a model-based variant of AlphaZero that has the same representation and prediction networks, but also learns a model of the dynamics and predicts rewards, which it uses for planning. Specifically, it learns a dynamics network *f*^dyn^ that predicts the next latent state ${{\bf{\text{h}}}}_{t}^{k+1}$ and reward ${\hat{r}}_{t}^{k+1}$ resulting from a transition. Note that the subscript *t* denotes timesteps in the real environment and the superscript *k* represents timesteps in the model.

1 $${{\bf{\text{h}}}}_{t}={f}^{rep}({{\bf{\text{S}}}}_{t})$$

2 $${{\bf{\text{h}}}}_{t}^{k+1},\,{\hat{r}}_{t}^{k+1}={f}^{dyn}({{\bf{\text{h}}}}_{t}^{k},{{\bf{\text{a}}}}_{t}^{k})$$

3 $${\hat{v}}_{t},\,{\hat{\pi }}_{t}={f}^{pred}({{\bf{\text{h}}}}_{t})$$

On reaching a new state, AlphaZero proceeds by first encoding the state into a latent representation with the representation network. Then, the true dynamics or dynamics network (for MuZero) as well as the prediction network *f*^pred^(**h**_*t*_) are used to simulate several trajectories that fill out a search tree, by sampling state transitions. At each node, the actions are selected using an optimistic strategy called the predictor upper confidence tree bound^32^, meant to balance exploration (trying new actions) and exploitation (progressing further down the subtree of the current estimate of the best action). This strategy starts out by following the predicted policy ${\hat{\pi }}_{t}$ closely, and gradually shifts towards maximizing the predicted value function. Ultimately, an action is recommended by sampling from the root node with probability proportional to its visit count during MCTS. The predicted policy is then trained to match the visit counts of the MCTS policy in an attempt to distil the search procedure into a policy such that subsequent iterations of MCTS will disregard nodes that are not promising.

#### Sorting networks

**Extended Data Fig. 2:**
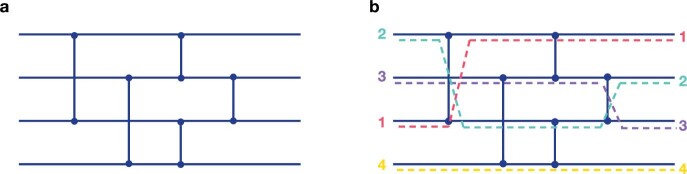
An example sorting network^43^. **(a)** The horizontal lines are called wires and the vertical lines are called comparators. **(b)** An initially unsorted sequence of values are input into the sorting network on the left hand side. At various stages two wires encounter a comparator. If the value at the top of the comparator is smaller than the value at the bottom of the comparator, the numbers switch wires. An optimal sorting network places comparators in specific positions so as to sort any sequence of unsorted values using the minimum number of comparators.

Sorting networks are very efficient as their structures can be parallelized on modern CPU architectures. They therefore tend to achieve faster runtime performance, especially on small sorts, compared to popular and efficient base case algorithms such as insertion sort^17,43,50^. A sorting network^43^ consists of two types of item called comparators (vertical lines) and wires (horizontal lines) (Extended Data Fig. 2a). Each wire carries a value from left to right. When two wires intersect at a comparator, the values on the two wires are compared. If the value of the bottom wire is smaller than the value of the top wire, then the values are swapped between wires as seen in Extended Data Fig. 2b. A programmatic implementation of a sorting network consists of executing these swaps on particular pairs of elements from the input sequence in a particular order.

### Action pruning rules

We pruned the action space by removing some program invariances (for example, the order of register allocation) and illegal instructions (for example, comparing two memory locations). This helps reducing the size of the action space and increases convergence rate. For our experiments, we used the following rules:

1. Memory locations are always read in incremental order.
2. Registers are allocated in incremental order.
3. We cannot compare or conditionally move to a memory location (illegal).
4. We can read and write to each memory location only once.
5. We cannot use non-initialized registers (illegal).
6. Do not perform consecutive compare instructions.

#### Training regime

We train AlphaDev on a Tensor Processing Unit (TPU) v.3, with a total batch size of 1,024 per TPU core. We use up to 16 TPU cores and train for 1 million iterations. On the actor side, the games are played on standalone TPU v.4, and we use up to 512 actors. In practice, across all tasks, training takes, in the worst case, 2 days to converge.

#### AlphaDev-S

It is important to understand the advantages and limitations of RL compared to other possible approaches for program optimization. As such, we implemented a state-of-the-art stochastic superoptimization approach^8^ and incorporated it into AlphaDev as the learning algorithm to optimize sorting functions. We refer to this adapted version as AlphaDev-S. Our re-implementation has been specifically optimized for the sorting domain. This includes implementing the algorithm to run with our assembly environment, defining a correctness and performance loss function specific to sorting and running extensive hyperparameter sweeps to identify the best variant. The cost function used for AlphaDev-S is *c* = correctness + *α* × performance where correctness corresponds to computing the number of incorrect input sequence elements that are still unsorted, performance corresponds to the algorithm length reward and *α* is a weight trading off the two cost functions. We are unable to optimize directly for latency as this slows down the learning algorithm considerably making learning infeasible. It should be noted that this function has been adapted to support the same set of assembly instructions used by AlphaDev as well as prune the same set of incorrect or illegal actions. It also uses the same program correctness computation module (Fig. 2b) to compute the correctness term.

AlphaDev-S is then executed by first proposing a transformation to the program stored in the buffer (which may be empty or initialized with an already sorted program). The correctness and performance terms are then computed using the program correctness module and algorithm length, respectively. If the cost is lower than the current best cost, the new program is accepted with high probability, otherwise it is rejected. We will now discuss the correctness cost function and transform weights in more detail.

#### Correctness cost

For the correctness cost function, we implemented three types of cost function. The first one is defined as the percentage of incorrectly placed items: $\frac{P-P{C}_{t}}{P}$ where *P* is the total number of items to place and PC_*t*_ is number of correctly placed items at timestep *t*. The second variant is the square root of this equation. The final cost function takes the square root of the difference $\sqrt{-{PC}_{t}}$ and this is what yielded the best performance.

#### Program transformations

We enabled several program transformations such as adding an instruction to increase the size of the program (Add Transform), swapping two instructions (Swap Transform), randomly changing an Opcode for an instruction (Opcode Transform), randomly sampling an Operand for a chosen instruction (Operand Transform) and randomly sample an Opcode and its corresponding Operands (Instruction Transform). It is possible to influence the sampling of these transforms to encourage some to be sampled more or less frequently. We optimized the weights for sampling transforms by running an extensive hyperparameter sweep.

### Investigative studies for AlphaDev variants

We now present a set of investigative studies that help to better understand the advantages and limitations of the DRL and the stochastic search learning algorithms used in AlphaDev. We compare AlphaDev to AlphaDev-S. We implemented two variants of AlphaDev-S: (1) Cold Start (AlphaDev-S-CS) and (2) Warm Start (AlphaDev-S-WS). AlphaDev-S-CS uses no previous information and has to generate a program from an empty program buffer. AlphaDev-S-WS’s buffer is warm started with a correct sorting program (for example, optimal sorting network assembly program) and it edits the program to optimize it further. We compared the variants with AlphaDev in both the individual and variable sort algorithm setups.

Because AlphaDev always learns from scratch with no previous knowledge, the direct comparison would be to the cold start stochastic search version: AlphaDev-S-CS. However, as initial near-optimal programs may sometimes be available, we also compare AlphaDev to the warm start stochastic search version: AlphaDev-S-WS.

It should be noted that the stochastic search variants are unable to optimize directly for latency, as this would make learning infeasible because of computational efficiency. As such, our AlphaDev-S variants optimize for algorithm length. Then, at the end of training, we iterate through the set of generated programs for AlphaDev-S across varying lengths and identify the program with the lowest latency.

In each case, the stochastic search algorithms (AlphaDev-S) are run using at least the same computational resources and wall-clock time to that of AlphaDev.

#### Fixed sort

We first examine the performance of the various approaches for the fixed sort algorithms. In this case, all algorithmic variants optimize for algorithm length as algorithm length and latency are highly correlated in the conditional branchless setting (see Supplementary Information for more details).

In the cold start setting, AlphaDev-S-CS is unable to find the optimal programs in each case as seen in Extended Data Table 2a. In addition, AlphaDev-S-CS explores orders of magnitude more programs than AlphaDev as shown in Extended Data Table 2b. In the warm start setting, AlphaDev-S is warm started with a near-optimal sorted program, and is able to match the performance of AlphaDev in each case as shown in Extended Data Table 2a. It is more computationally efficient than AlphaDev as shown in Extended Data Table 2c but explores orders of magnitude more programs for sort 3 and sort 5 as shown in Extended Data Table 2b. It can be argued that AlphaDev-S-WS has a substantial advantage in this scenario as it is provided with an initial near-optimal program. We will show in the Variable sort section that when the algorithms become more complicated and branching is introduced, warm starting the learning algorithm with a near-optimal program is not enough and can cause it to get stuck in suboptimal solutions.

#### Brute-force approach

We also used a brute-force approach to prove that no program shorter than 17 instructions exists for sort 3. We had to enumerate roughly 10^32^ programs and, even with pruning heuristics, it took more than 3 days to prove this hypothesis. For sort 4 and above this approach is infeasible.

#### Latency benchmarking suite

The length of a program is only a proxy for the performance of an algorithm. As we introduce branching structures, the length and latency of a program are not well correlated. Therefore, we run the programs on actual machines and measure their latency. Microbenchmarking is very challenging given the numerous noise sources that could affect the measurements. This is especially true when running on shared machines where there could be interference from other processes. Our approach is to have a separate benchmarking service, replicated on separated machines, so that we can quickly perform many measurements in a controlled environment under different conditions. The system works as follows:

1. The RL agent processes 1,000 measurements across the machines using the replicated service.
2. For each measurement, the service runs the given sorting algorithm over 10,000 random inputs (for example, for sort 3 this would be 3 × 10,000 = 30,000 random integers).
3. We measure the time taken using a CPU performance counter (CPU_CLK_UNHALTED.CORE).

We then take the fifth percentile as our final measurement, because we assume that most noise sources are one-sided (for example, cache misses, pre-emptions and so on). During training we process the measurements across ten machines for computational efficiency. After training, we benchmark AlphaDev’s solution against the baseline solutions, and process the measurements across 100 machines for more accuracy and noise reduction. For each benchmark, we compute confidence intervals using the distribution-free two-sided confidence interval for a quantile tabular method^44^.

#### Variable sort

When optimizing directly for latency, AlphaDev outperforms AlphaDev-S-WS on VarSort3, VarSort4 and VarSort5 as seen in Extended Data Table 3a. AlphaDev-S-CS fails to find a solution in each case. In the cases of VarSort4 and VarSort5, program length and latency are not correlated (see Supplementary Information for more details). This indicates that when program length cannot be used as a proxy for performance, AlphaDev is able to find lower latency solutions compared to AlphaDev-S. This is even in the case where the stochastic search is warm started with a near-optimal program. In addition, AlphaDev converges to the optimal solution after exploring a maximum of 12M programs as seen in Extended Data Table 3b. This is orders of magnitude lower than that of AlphaDev-S-CS and AlphaDev-S-WS, respectively (31 trillion programs in the worst case).

### Exploration hypothesis

**Extended Data Fig. 3:**
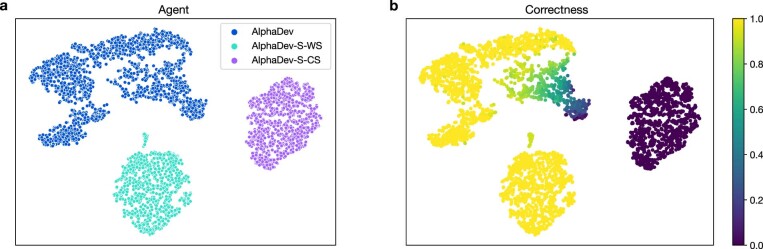
Hypothesis for improved exploration using AlphaDev. **(a)** A 2D t-SNE^51^ projection indicating the regions explored by AlphaDev (blue) compared to AlphaDev-S. **(b)** The same 2D t-SNE projection as in (a) with algorithm correctness superimposed onto each point from incorrect programs (purple) to correct programs (yellow). As seen in the figure, AlphaDev-S struggles to move out of local optima whereas AlphaDev is able to explore from the space of incorrect programs to the space of correct programs.

We proposed that AlphaDev-S struggles to discover programs when learning from scratch and gets stuck in local optima when warm started because of its limited exploration capabilities as a result of the stochastic search procedure. Extended Data Fig. 3 shows two-dimensional *t*-stochastic neighbour embedding (*t*-SNE) projections^51^ of AlphaDev and AlphaDev-S’s assembly algorithms discovered during their respective training procedures for VarSort5. The features used in the projection include correctness, latency, algorithm length and a histogram count of the instructions used per algorithm. Extended Data Fig. 3a indicates the regions in algorithm space explored by AlphaDev, AlphaDev-S-CS and AlphaDev-S-WS, respectively, whereas Extended Data Fig. 3b superimposes algorithm correctness onto each point in the *t*-SNE projection in which the colour indicates the correctness of each discovered algorithm, ranging from incorrect algorithms (purple) to correct algorithms (yellow). The AlphaDev-S variants both cover a densely packed circular region around their initial seed, which highlights the breadth-first nature of their stochastic search procedure. This illustrates that AlphaDev-S-CS fails to navigate through the space of incorrect algorithms in a reasonable amount of time and discover correct algorithms when learning from scratch. A similar argument applies to AlphaDev-S-WS whereby, when optimizing from an already correct but suboptimal expert demonstration, the algorithm is biased towards exploring its vicinity and struggles to escape this local maxima. By contrast, AlphaDev has more diverse algorithm space coverage, as the long-term value function is a guiding signal for discovering new and interesting parts of algorithm space. As seen in Extended Data Fig. 3b, it is capable of escaping the space of incorrect algorithms to discover a new space of correct algorithms, highlighting the exploration advantages afforded by AlphaDev.

### Related work

#### Assembly optimization

There are numerous approaches to optimizing assembly programs, which we have classified into three groups: enumerative search, stochastic search and symbolic search^5^.

First, enumerative search techniques include brute-force program enumeration^4–6^ as well as implicit enumeration using symbolic theorem proving^52,53^. These approaches search through the space of programs to find a solution based on a predefined set of programs, heuristic and/or cost function. These approaches struggle to span large regions of program space, especially as the size and complexity of the program increases.

Second, stochastic search techniques circumvent comprehensive enumeration by relying on sampling mechanisms such as Markov chain Monte Carlo sampling^5,6,8,9^. Rajeev Alur et al.^5^ define a correctness specification, provided by a logical formula that uses symbols from a background theory. The goal is to then find an implementation expression such that logical formula defining the specification is valid. The idea is to iteratively add test cases and then search and expand the program to solve the given test cases. They optimize for correctness on problems from the book Hacker’s delight^54^. Phitchaya Mangpo Phothilimthana et al.^6^ introduce the LENS algorithm that is based on running enumerative, stochastic and symbolic search in parallel, while relying on handcrafted pruning rules. This setup is capable of optimizing up to 21 instructions, and cannot optimize for latency nor support branching. Another algorithm^8^ is based on Markov chain Monte Carlo rejection sampling and applies transformations to programs in assembly using a loss function that is a function of correctness and performance. Many of these approaches are prone to getting stuck in local minima and may also struggle as the size and/or complexity of the program increases. In addition, incorporating actual, measured latency into these approaches are either infeasible or prohibitively expensive.

Third, symbolic search approaches can also be implemented to optimize assembly programs. These include SAT solvers^55^, SMT solvers^5,6^ and Mixed Integer Programs (MIPs)^56,57^. However, these approaches suffer from scaling issues. For example, classical solvers require a problem to be translated into a certain canonical form. It usually requires an expert in the said solvers and a substantial amount of time to find an efficient formulation. In addition, for any new modification of the problem, this has to be repeated. Classical solvers are also hard to parallelize and thus, it is challenging to leverage more hardware to speed up the solving process. Another symbolic search algorithm is Cholorphyll^10^ that implements a multi-phase approach. It first requires as input a source program with partition annotations that specify where code and data reside. Then, a layout synthesizer maps program fragments onto physical cores to minimize computational costs. The code is then separated into per-core program fragments and the program fragments are compiled into machine code. At this point, a superoptimizer optimizes each of these fragments.

#### SIMD optimization

Various approaches^58–60^ have also been applied to sorting functions that run in the single instruction, multiple data (SIMD)^61^ setup. This setup is capable of parallelizing instruction execution, but is not supported at present in popular libraries such as LLVM’s libc++ std::sort library. One example is that from Gilles Barthe et al.^7^ that proposes a methodology for optimizing programs by automatically vectorizing loops with SIMD instructions. They do this by introducing a framework for verifying the correctness of transformations to a program and performing a search-based procedure using the said transformation. Their framework can discover SIMD looping structures of up to nine instructions in 0.12 s, which corresponds to a minimum 2× speed-up.

#### RL approaches for program synthesis

There are also several studies using RL for program optimization. Kevin Ellis et al.^62^ learn a policy and value function to write and evaluate code, as well as performing a Monte Carlo-style search strategy during inference. This work requires a pretraining step and aims to generate correct programs that satisfy a predefined specification. The approach is successfully applied to computer-aided design and string editing programs. SuperSonic^63^ uses an RL meta-optimizer to select between different RL architectures, using a Multi-Armed Bandit policy search to find a state representation, reward function and RL algorithm that is optimal for the current task. This requires keeping track of many RL algorithms and architectures, which are used as part of the state space. By contrast, our approach only focuses on training a single RL architecture, taking advantage of MCTS search and powerful state representations. Shypula et al.^64^ create a supervised assembly dataset and use it to train a Transformer model for mapping unoptimized to optimized code, followed by an RL stage for improving the solution quality. Our method does not require a supervised dataset or two separate training and finetuning stages, and optimizes everything end-to-end using RL and search instead. Chen et al.^65^ define their own domain specific language and perform input–output program synthesis that better uses the intermediate program representation to guide the synthesis routine. They show that this can be incorporated with RL, using the setup of Rudy Bunel et al.^66^ and improve the correctness of generated functions. They do not, however, optimize for program length or latency.

#### Input–output examples for program synthesis

A large body of work addresses the problem of learning programs from input–output pairs. One type of approach learns a neural network for matching inputs to outputs directly^11,13,67,68^. This approach is difficult to integrate into existing libraries and can struggle to generalize to previously unseen inputs, although there has been some encouraging recent progress using graph representations^69^. Another type of approach is to perform a search in program space, guided by a learned model^12,70–72^. For instance, Chen et al.^70^ use a model that predicts the next program token on the basis of a partial program and the input–output pairs. This bears some similarities to how search is guided in our approach: the learned policy prior in AlphaZero is a model for predicting the next token, learned on the basis of a combination of a partial program and that program’s effects on the inputs. However, we are interested in finding correct and efficient programs, which we achieve by further learning a value function for approximating the expected latency of partial programs, and using AlphaZero to incorporate this value function into the search process.

#### Deep learning for code generation

There are also several deep learning approaches that use large languages models to generate code. These approaches vary in their uses from transpilation, code refactoring and explaining code^15^ to generating human-level competitive code using a natural language description^14^. That particular work aims to generate correct code, but does not focus on generating low-latency solutions.

#### Sort-based program optimization

There are several program synthesis studies that have tackled sorting algorithms. For example, White et al.^26^ use RL for learning sorting functions. Their work uses several heuristics and a domain specific language to yield a sorting algorithm called reinforcement programming sort. Srivastava et al.^27^ encodes the program synthesis as a verification problem. Specifically, they represent a synthesis task as a tuple consisting of the functional expression, the domains and guards appearing in the synthesized program and the resource constraints. The idea is that, given a prespecified resource constraint, their synthesizer produces a program that meets the predefined specification to ensure correctness. They apply this to discover merge sort and quick sort. Jason Ansel et al.^28^ takes as input predefined algorithms (for example, insertion sort, merge sort and quick sort) and then determines when to select these algorithms for execution using its autotuner function. It does so by defining a language that contains rules and transforms that dictate how the algorithms are selected and where they are executed.

## Online content

Any methods, additional references, Nature Portfolio reporting summaries, source data, extended data, supplementary information, acknowledgements, peer review information; details of author contributions and competing interests; and statements of data and code availability are available at 10.1038/s41586-023-06004-9.

### Supplementary information

Supplementary Information

## Figures and Tables

**Extended Data Table 1:**
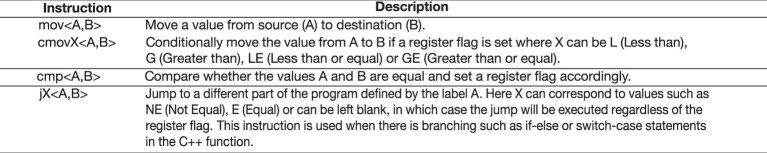
Additional Assembly instructions

This table contains a list of additional assembly X86 instructions using AT&T syntax and their corresponding description.

**Extended Data Table 2:**
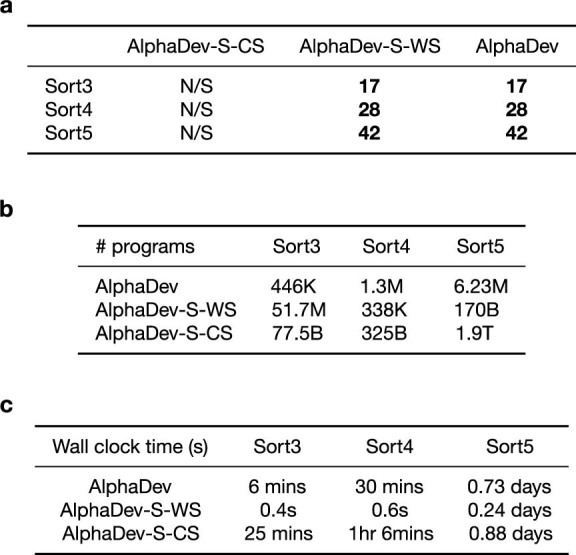
Comparison of AlphaDev and AlphaDev-S for fixed sort

(**a**) Presents the shortest programs found by each approach. Note that AlphaDev-S-CS is unable to discover a sorting function when training from scratch. AlphaDev-S-WS, which is initialized with a near-optimal program, is able to match the performance of AlphaDev, which discovers the optimal programs from scratch. (**b**) Indicates the number of programs explored by each approach to find the optimal solution. Note that AlphaDev-S-CS explores orders of magnitude more programs for each sort algorithm. For sort 3 and sort 5 AlphaDev-S-WS explores orders of magnitude more programs than AlphaDev to find the optimal solution. (**c**) The approximate wall clock time to generate the shortest program for each sort length. AlphaDev-S-WS is more computationally efficient than AlphaDev for branchless sort. However, as will be shown in Extended Data Table 3, when branching is introduced, AlphaDev outperforms AlphaDev-S-WS, which tends to get stuck in locally optimal solutions.

**Extended Data Table 3:**
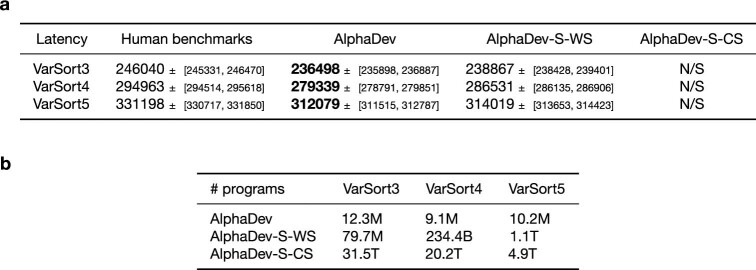
Comparison of AlphaDev and AlphaDev-S on variable sort

(**a**) Presents the latency results for the programs discovered by each approach. The reported latency corresponds to the 5^*th*^ percentile of latencies measured across 100 machines. The ± [Lower, Upper] reports the lower and upper confidence intervals respectively. In this setting, AlphaDev optimizes directly for real, measured latency. Note that AlphaDev outperforms each approach and AlphaDev-S-CS is unable to find a solution in each case. (**b**) In the variable sort setting, both AlphaDev-S variants explore orders of magnitude more programs compared to AlphaDev.

## Data Availability

The data used to train the system were generated synthetically according to the procedures explained in the paper. The algorithms discovered by AlphaDev for the copy and swap operators are presented in the main paper. We have also released the discovered AlphaDev assembly implementations for sort 3–8 as well as VarSort3, 4 and 5 on Github at https://github.com/deepmind/alphadev. We have included exhaustive tests to ensure that each implementation is correct. In addition, Appendix G in Supplementary Information contains a list of additional, correct sorting algorithms discovered by AlphaDev for sort 3, sort 4 and sort 5. The performance of the sort 3, sort 4 and sort 5 algorithms on the official LLVM benchmarking suite for three different CPU architectures as well as floats, int32 and int64 data types is detailed in Appendix E in the Supplementary Information. In addition, the AlphaDev sort 3, sort 4 and sort 5 implementations can be found in the LLVM libc++ standard sorting library^3^.

## Code availability

We have also released pseudocode at https://github.com/deepmind/alphadev that includes the environment, the full actor and training loops as well as the core MCTS algorithm. In addition, we include our actual JAX implementation of our policy, value and representation networks that enable the architectures to be reproduced. Finally, we have a config file containing the hyperparameter definitions to be used with the agent.
