# Supplementary material for: Faster sorting algorithms discovered using deep reinforcement learning
Source: Nature. 2023 Jun 7;618(7964):257–63. doi: 10.1038/s41586-023-06004-9 (PMC10247365; doi:10.1038/s41586-023-06004-9)
Supplement: Supplementary file 1 — Supplementary Information [file 41586_2023_6004_MOESM1_ESM.pdf]

nature portfolio

https://doi.org/10.1038/s41586-023-06004-9

Supplementary information

Faster sorting algorithms discovered using deep reinforcement learning

In the format provided by the authors and unedited

Faster sorting algorithms discovered using deep reinforcement learning (Supplementary Information)

Daniel J. Mankowitz[1,3,✉] Andrea Michi[1,3] Anton Zhernov[1,3] Marco Gelmi[1,3]
Marco Selvi[1,3] Cosmin Paduraru[1,3] Edouard Leurent[1,3] Shariq Iqbal[1]
Jean-Baptiste Lespiau[1] Alex Ahern[1] Thomas Köppe[1] Kevin Millikin[1]
Stephen Gaffney[1] Sophie Elster[1] Jackson Broshear[1] Chris Gamble[1]
Kieran Milan[1] Robert Tung[1] Minjae Hwang[2] Taylan Cemgil[1]
Mohammadamin Barekatain[1] Yujia Li[1] Amol Mandhane[1] Thomas Hubert[1]
Julian Schrittwieser[1] Demis Hassabis[1] Pushmeet Kohli[1] Martin Riedmiller[1]
Oriol Vinyals[1] David Silver[1]

Contents

A Hyperparameters **1**

B Correlation between algorithm length and latency **3**

C Scaling to larger sorts **3**
C.1 Graph neural network encoder 5

D Additional domains **6**
D.1 VarInt Serialization and Deserialization functions 6
D.2 Competitive coding 7

E LLVM patch **7**

F Theorem: the Assembly Zero-One Principle **11**
F.1 Assembly Sorting Network 11
F.2 Assembly Zero-One Principle 12
F.3 Proof of Theorem F.1 14
F.4 Proof of Theorem F.2 16

G Additional programs **17**

A Hyperparameters

In the experiments, for *AlphaDev*, we mostly used the same neural network architecture, optimisation and hyperparameters used by AlphaZero [1]. The main differences are the architecture used for the representation and prediction

[1]Deepmind, London, UK.
[2]Google, Mountain View, CA, USA.
[3]These authors contributed equally
✉Corresponding author: dmankowitz@deepmind.com

networks, which we define in Table A.1 and A.2 respectively. In addition the hyperparameters for *AlphaDev-S-WS* and *AlphaDev-S-CS* can be found in Tables A.3 and A.4 respectively.

Module	Hyperparameters	Representation Network
Transformer Encoder	Architecture	MultiQuery Transformer Encoder
	Number of layers	6
	Number of heads	4
	Output size	128
CPU State Encoder	Architecture	MLP
	Number of layers	2
	Embedding dimension	512
	Activation function	relu

Table A.1: Hyperparameters for the Representation Network. The MultiQuery Transformer encoder uses a form of multi-head attention with multiple (in our case 4) *query* heads while using only one key and value head.

Head	Hyperparameters	Prediction Network
Value Correctness/Latency	Architecture	MLP
	Embedding dimension	512
	Number of layers	2
	Output distribution	Categorical [2]
	Head loss	Categorical cross entropy
Policy	Architecture	MLP
	Embedding dimension	512
	Number of layers	2
	Output size	Number of actions
	Head loss	Softmax cross entropy

Table A.2: Hyperparameters for the Prediction Network. This includes the hyperparameters for the dual value function correction and latency heads as well as for the policy.

AlphaDev-S-WS Hyperparameters	Values for sort<3,4,5,6,7,8>	Values for VarSort<3,4,5>
α	<0.05, 0.05, 5.0, 0.01, 0.1, 0.01>	<0.01, 1.5, 1.0>
Beta (Annealing constant)	<100, 100, 10, 100, 30, 0.1>	<30, 100, 30>
instruction weight	1	1
Swap weight	1	1
Opcode weight	1	1
Operand weight	<1,1,3,1,1,3>	<1, 3, 1>
Add weight	0	0

Table A.3: *AlphaDev-S-WS* Hyperparameters. Note that the optimal hyperparameters were identified after running an extensive hyperparameter sweep.

AlphaDev-S-CS Hyperparameters	Values for sort<3,4,5,6,7,8>	Values for VarSort<3,4,5>
α	<0.01, 0.01, 0.01, 0.1, 0.01, 0.01>	< 1.0, 1.5, 0.01 >
Beta (Annealing constant)	<100, 100, 100, 30, 100, 100>	< 30, 100, 30>
instruction weight	1	1
Swap weight	1	1
Opcode weight	1	1
Operand weight	<1,1,1,1,1,1>	<1,3,1>
Add weight	0	0

Table A.4: *AlphaDev-S-CS* Hyperparameters. Note that the optimal hyperparameters were identified after running an extensive hyperparameter sweep.

B Correlation between algorithm length and latency

It is critical in our branchless conditional assembly setup that if we use algorithm length as a proxy for latency, then there should be a strong correlation between the two. To measure this correlation, we ran *AlphaDev-S* and generated correct sorting programs over a range of lengths for both fixed and variable sort algorithms. We then measured the latency of each program using the latency benchmarking suite (see Methods for more details) and computed the Pearson correlation between algorithm length and latency.

Fixed sort. The correlation for each fixed sort algorithm is shown in the first five columns of Table B.5. For sort8, not enough data points were generated to compute correlation, but we assume that the correlation holds here too since the program structures are still branchless conditional assembly and do not drastically change between sorting programs. As seen in the table, there is strong positive correlation for each task which implies that algorithm length is indeed a good proxy for latency in this setup. The full correlation plots are presented in Figure B.1.

Task	Sort3	Sort4	Sort5	Sort6	Sort7	VarSort3	VarSort4	VarSort5
Pearson correlation of length vs. latency	1.00	1.00	0.98	0.99	0.98	0.65	-0.02	-0.68

Table B.5: The pearson correlation coefficient between algorithm length and latency for fixed and variable sort algorithms discovered by *AlphaDev-S*. Note that for the conditional branchless algorithms (sort3 to sort7), there is strong correlation between length and latency. However, for variable sort algorithms that contain branching, the correlation breaks down as the size of the sort algorithm increases.

Variable sort. In the variable sort algorithm setting, additional algorithm complexity in the form of branching is introduced. This factor largely removes the correlation between algorithm length and latency. The last three columns in Table B.5 present the Pearson correlation between algorithm length and latency for each of the variable sort functions. As seen in the table, as the size of the sort increases, the correlation breaks-down. The correlation plots corresponding to this table are presented in Figure B.2. This also indicates that, in the variable sort case, we want to optimize directly for latency rather than algorithm length, to find optimal low-latency algorithms.

C Scaling to larger sorts

While sort3 to sort5 are fundamentally important algorithms, we investigate approaches to scaling to larger fixed sorting algorithms. As such, we focused on solving sort6 to sort8 using *AlphaDev*. The main bottleneck for scaling is the increasing number of sequences that need to be computed to determine algorithm correctness. For example, sort6 and sort7 require testing 4683 and 47293 sequences for correctness respectively. We incorporate three modifications that help improve the capabilities of *AlphaDev*. First, we address the increasing number of sequences for correctness by extending the well-known zero-one principle [3, 4] to our setting. We refer to this as the Assembly zero-one principle

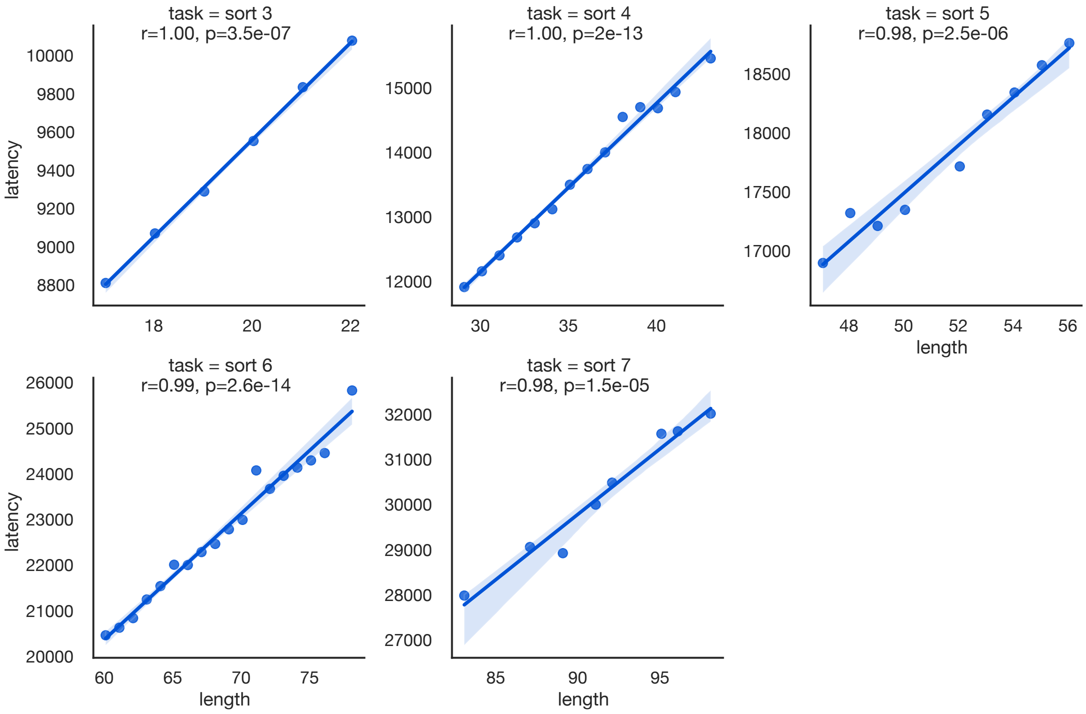

Figure B.1: Pearson correlation of Algorithm length *vs.* latency for fixed sort algorithms generated by *AlphaDev-S*. Note the strong correlation between length and latency for the conditional branchless sorting algorithms.

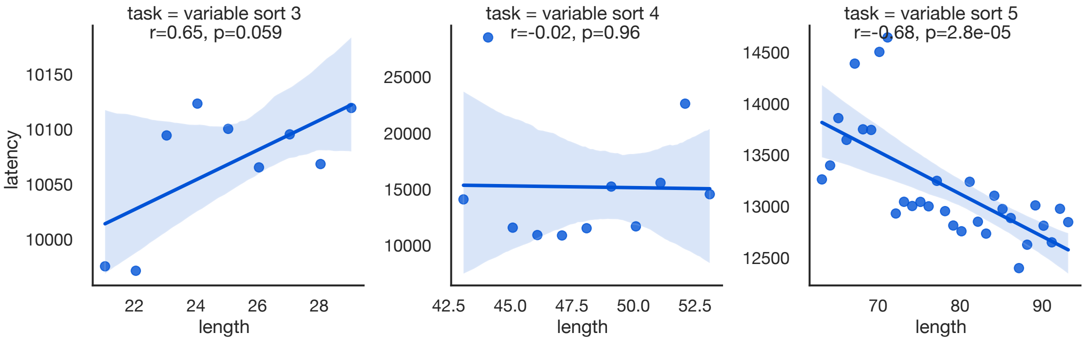

Figure B.2: Pearson correlation of Algorithm length *vs.* latency for variable sort algorithms generated by *AlphaDev-S*. As seen in the figure, as the length of the variable sort algorithm increases, the correlation between length and latency breaks down.

theorem; see Appendix F for the full proof. Our theorem states that it is sufficient to test fixed sorting algorithms on all *binary* sequences to ensure algorithm correctness. As such, sort6 and sort7 algorithms only need to be tested on $2^6 = 64$ and $2^7 = 128$ sequences respectively. Second, we utilize a Graph Neural Network (GNN) [5] in place of the Transformer Encoder in the *AlphaDev* representation network. The GNN is well suited for fixed sort algorithms (i.e., branchless assembly) but requires significant modifications to capture the structure of branched assembly programs which we leave for future work; see Appendix C.1 for a full overview of the GNN. In addition, we utilize MuZero [6] as the *AlphaDev* learning algorithm. This is to primarily improve the speed of the agent as it uses a learned model during MCTS. We refer to this variant as *AlphaDev-M*. Finally, we use re-analyze [7] which uses sub-optimal, but correct demonstrations to help guide the search of the agent. *AlphaDev-M* improves upon the state-of-the-art human

benchmarks for sort6, sort7 and sort8 by reducing the number of instructions by 3, 2 and 1 respectively.

C.1 Graph neural network encoder

As mentioned in the main paper, the representation $\mathbf{P_t}$ of the algorithm generated thus far can benefit from capturing the algorithmic structure. Graph-based representations have been shown to improve performance in tasks such as program classification and induction [8] or variable naming and identifying variable misuse [9]. Such results are an indication that the inductive biases and invariances incorporated by graph neural networks are effective in building models that learn efficiently and generalize in algorithm space. As such, we introduce our Graph Neural Network [5, 10, 11] architecture that models the structure of assembly programs. As seen in Figure C.3, we define a graph where nodes (circles) represent memory and register locations as well as register flags (e.g., 'Flag' node), and edges (arrows) correspond to data flow induced by instructions. These nodes store as their features the values of the corresponding locations after executing the program on all possible inputs. Edge features include the instruction order (e.g., a, b, c, d), instruction arguments and the instruction to be executed. After *AlphaDev* executes an action, an instruction is appended to the graph via an edge. Global features are also included that consist of a single vector summarizing information about the entire graph. For example, an input global feature could be the algorithm length. The hyperparameters used in the GNN implementation are found in Table C.6.

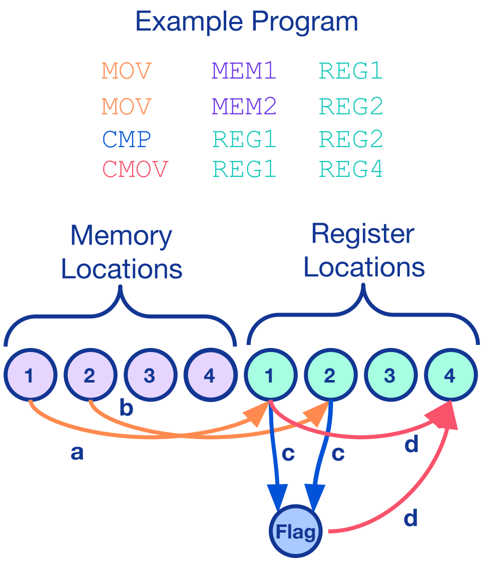

Figure C.3: GNN representation of assembly programs. The GNN representation consists of nodes (circles) that correspond to memory and register locations as well as register flags. The edges (arrows) correspond to data flow induced by instructions. Edge features include instruction order (e.g., a, b, c, d), instruction operands and the instruction to be executed. The GNN network can be used interchangeably with the Transformer Encoder module in Extended Data Figure 1.

Module	Hyperparameters	GNN
Graph Neural Network	Number of blocks	1
Global update	Architecture	Residual blocks
	Number of blocks	4
	Number of layers per block	2
	Embedding dimension	512
Node update	Architecture	Residual blocks
	Number of blocks	4
	Number of layers per block	2
	Embedding dimension	512
Edge update	Architecture	Residual blocks
	Number of blocks	4
	Number of layers per block	2
	Embedding dimension	512
Edge Aggregation	Type	Sum

Table C.6: Hyperparameters for the GNN representation network.

D Additional domains

D.1 VarInt Serialization and Deserialization functions

Data stored using Protocol Buffer format is serialized before transmission. The receiving end can then deserialize the data. The VarInt algorithm [12] is a key component in both the serialization and deserialization process. It is also one of the main contributors to computational costs during deserialization. The human benchmark, which is the current open-source implementation for the VarInt deserialization function, contains branching (i.e., if-else statements) and needs to deserialize a binary input sequence of 5 bytes in length.

A VarInt serialization algorithm receives as input a value (e.g., 300) and serializes this value into a sequence of bytes. The serialized bytes are then transmitted over a specified protocol. A VarInt decoding algorithm receives as input the sequence of binary bytes and deserializes this back to the original value (i.e., 300). A more detailed example of a VarInt serialization/deserialization process is detailed below:

Varint Serialization routine.

- A value, for example 300 is fed as input into the VarInt serialization routine.
- This value is then serialized into a binary sequence of bytes. In this example, we need two bytes to represent 300 as: 10101100 00000010.

Varint Deserialization routine. The serialized representation can be transformed back into 300 as follows:

- Drop the most significant bit for each byte to yield: 0101100 0000010.
- As the VarInt encoding stores numbers with the least significant group first, we reverse the order of each byte to yield: 0000010 0101100 which corresponds to the binary representation of 300

The VarInt Deserialization planning problem. When training an agent to generate the VarInt deserialization routine, there are various planning considerations to be made. For example, the Most Significant Bit (MSB) in each byte of the representation is critical to successfully deserialize the serialized value. The MSB indicates whether the subsequent byte should be read. For example, the two byte serialized representation in the above example 10101100 00000010 indicates that the second byte should be read as the first byte's MSB is set to 1. However, the second byte's MSB is 0

indicating that, if subsequent bytes exist in the serialized sequence, they should not be read. Moreover, as numbers are stored with the least significant group first, the order of the bytes need to be reversed efficiently. It is therefore critical to construct the VarInt de-serialization algorithm such that it captures this relationship and finds optimized routines to make the function more efficient.

VarInt assignment move. When deserializing data byte-by-byte, the agent needs to decide whether it should decode the next byte or not. The most significant bit in each byte is called the continuation bit. If it is set to 1 then the next byte should be decoded; if it is set to 0 then the next byte should not be decoded and the decoding is finished. Typically, an instruction is required to test the continuation bit to determine whether it is set or not, and then set a flag, and a second instruction is required to reset the continuation bit to zero. The *AlphaDev* agent discovered a way to combine these two operations into a single instruction by making using of the `add<A,B>` instruction – adds the value in A to the value in B – in a clever way. We refer to this as the *Varint assignment move*. The instruction is defined as `add` < $-2^{7\times B}$, `RegisterX` > where B refers to the number of bytes that have been processed thus far and are stored in RegisterX. By adding this to RegisterX, it has the effect of (1) setting a flag if the continuation bit is set and (2) resetting that bit to zero. Subsequent instructions then make use of the flag value.

D.2 Competitive coding

We selected a competitive programming problem (*Buttons* [13]) from the *CodeContests* dataset [14] and used a correct human solution from the dataset as the benchmark. We defined an algorithm correctness reward and a latency reward as in previous domains. To verify correctness, we generate an exhaustive set of input, output pairs that *AlphaDev* uses for evaluating the generated programs whilst playing the game. Using *AlphaDev*, the agent is able to discover a lower latency algorithm than the human benchmark as shown in Table 1b. Interestingly, the lower latency program in this case is a longer program (29 instructions) compared to the human benchmark (13 instructions). The agent has found an efficient way to replace expensive multiplication operations with addition and subtraction instructions. This further highlights the need to optimize directly for latency as opposed to program length.

E LLVM patch

Using the official libc++ benchmarking suite, the performance of the sort3, sort4 and sort5 *AlphaDev* implementations compared to the original libc++ implementation are presented in the Tables below. The performance is computed across three different CPU architectures: Intel Skylake, ARMv8 and AMD Zen 2 for three different data types, namely: int32 (Table E.7), int64 (Table E.8) and floats (Table E.9). The columns for each Table are setup as follows: *sortK* indicates the number of elements being sorted by both algorithms. For example, sort1024 benchmarks the speed of sorting 1024 elements using the libc++ sorting library functions. As these functions call sort3, sort4 and sort5 multiple times, we are able to measure the efficiency gains for our approach on this large sorting function. The next column *original LLVM sort* contains the run-time performance of the *sortK* algorithm using the original LLVM sort3, sort4 and sort5 functions, measured in nano-seconds (ns). The *AlphaDev* column presents the run-time performance of the *sortK* algorithm using the *AlphaDev* sort3, sort4 and sort5 implementations. The next column, *change*, indicates the percentage change when comparing the original LLVM sort performance with the *AlphaDev* performance. Here, a negative percentage change indicates that *AlphaDev* outperforms the original LLVM sort algorithms. The final column contains the p-value which corresponds to the statistical significance of the result. Note that there are small regressions in performance for sort1 on ARMv8 for `uint32` and Intel Skylake for `uint64`. This is due to noise as the program simply terminates in this case and the latency that needs to be measured is negligibly small (less than a nanosecond).

cpu	sort$K$	original LLVM sort	*AlphaDev*	% change	p-value
Intel Skylake	1	3.98ns ± 5%	3.69ns ± 7%	-7.11%	0.000
	3	4.86ns ± 6%	2.18ns ± 5%	-55.18%	0.000
	4	5.43ns ± 8%	1.96ns ± 8%	-63.93%	0.000
	5	6.79ns ± 8%	1.98ns ± 7%	-70.87%	0.000
	16	10.5ns ± 9%	9.5ns ± 9%	-9.11%	0.000
	64	18.4ns ±13%	17.4ns ±11%	-5.69%	0.000
	256	25.8ns ±10%	24.7ns ± 8%	-4.09%	0.000
	1024	32.8ns ± 8%	31.9ns ± 9%	-2.79%	0.000
	16384	47.2ns ± 7%	46.3ns ±10%	-1.90%	0.000
	262144	61.4ns ± 7%	60.8ns ± 9%	-0.97%	0.000
ARMv8	1	3.85ns ± 0%	4.01ns ± 0%	+3.95%	0.000
	3	4.31ns ± 0%	2.11ns ± 0%	-51.04%	0.000
	4	4.83ns ± 0%	2.05ns ± 0%	-57.50%	0.000
	5	5.52ns ± 0%	2.15ns ± 0%	-61.01%	0.000
	16	9.59ns ± 0%	9.15ns ± 0%	-4.59%	0.000
	64	16.2ns ± 0%	15.7ns ± 0%	-2.99%	0.000
	256	22.3ns ± 1%	21.7ns ± 0%	-2.77%	0.000
	1024	28.5ns ± 0%	27.7ns ± 0%	-2.64%	0.000
	16384	40.3ns ± 1%	39.4ns ± 1%	-2.17%	0.000
	262144	51.8ns ± 2%	50.9ns ± 2%	-1.69%	0.000
AMD Zen 2	1	3.38ns ± 1%	3.38ns ± 1%	$\sim$	0.616
	3	4.45ns ± 1%	1.77ns ± 1%	-60.21%	0.000
	4	5.21ns ± 1%	1.57ns ± 1%	-69.85%	0.000
	5	5.87ns ± 5%	1.78ns ± 1%	-69.68%	0.000
	16	9.15ns ± 1%	8.90ns ± 0%	-2.65%	0.000
	64	16.6ns ± 1%	16.4ns ± 0%	-0.65%	0.000
	256	24.1ns ± 1%	24.0ns ± 1%	-0.40%	0.000
	1024	31.6ns ± 1%	31.3ns ± 1%	-0.71%	0.000
	16384	46.6ns ± 1%	46.2ns ± 1%	-0.81%	0.000
	262144	61.1ns ± 2%	60.8ns ± 3%	$\sim$	0.103

Table E.7: Performance of the *AlphaDev* **C++** Sort3,4,5 implementations on type **uint32**. Performance is benchmarked using the official LLVM microbenchmark. Note that the improved sort3, sort4 and sort5 routines discovered by *AlphaDev* improves the overall performance of significantly larger sorting functions (e.g., sort262144). This is because larger sorting algorithms repeatedly call small sort algorithms using divide-and-conquer approaches.

cpu	sort$K$	original LLVM sort	*AlphaDev*	% change	p-value	runs
Intel Skylake	1	3.68ns ± 5%	3.71ns ± 6%	+0.80%	0.000	
	3	4.95ns ± 8%	2.18ns ± 5%	-56.01%	0.000	
	4	5.39ns ± 7%	1.96ns ± 5%	-63.63%	0.000	
	5	6.91ns ± 6%	2.01ns ± 6%	-70.97%	0.000	
	16	10.0ns ± 9%	9.4ns ± 8%	-5.97%	0.000	
	64	17.9ns ±10%	17.3ns ± 9%	-3.03%	0.000	
	256	25.3ns ±10%	24.7ns ± 8%	-2.50%	0.000	
	1024	32.2ns ± 6%	31.6ns ± 6%	-1.80%	0.000	
	16384	46.9ns ± 7%	46.0ns ± 8%	-1.81%	0.000	
	262144	61.3ns ± 6%	60.5ns ± 9%	-1.27%	0.000	
ARMv8	1	4.02ns ± 0%	3.93ns ± 2%	-2.32%	0.000	
	3	4.36ns ± 0%	2.23ns ± 2%	-48.74%	0.000	
	4	5.03ns ± 0%	2.18ns ± 0%	-56.68%	0.000	
	5	5.61ns ± 0%	2.29ns ± 1%	-59.10%	0.000	
	16	9.63ns ± 0%	9.22ns ± 0%	-4.32%	0.000	
	64	16.2ns ± 0%	15.9ns ± 0%	-2.18%	0.000	
	256	22.4ns ± 0%	22.1ns ± 0%	-1.49%	0.000	
	1024	28.4ns ± 0%	28.0ns ± 0%	-1.16%	0.000	
	16384	40.0ns ± 1%	39.7ns ± 1%	-0.81%	0.000	
	262144	51.6ns ± 2%	51.4ns ± 2%	-0.48%	0.000	
AMD Zen 2	1	3.39ns ± 1%	3.39ns ± 0%	-0.19%	0.008	
	3	4.47ns ± 2%	1.77ns ± 1%	-60.42%	0.000	
	4	5.20ns ± 1%	1.56ns ± 1%	-69.88%	0.000	
	5	6.07ns ± 4%	1.59ns ± 1%	-73.73%	0.000	
	16	10.1ns ± 1%	9.0ns ± 1%	-10.80%	0.000	
	64	18.1ns ± 1%	16.6ns ± 1%	-8.45%	0.000	
	256	25.8ns ± 1%	24.2ns ± 1%	-6.16%	0.000	
	1024	33.2ns ± 1%	31.7ns ± 1%	-4.78%	0.000	
	16384	48.2ns ± 1%	46.7ns ± 1%	-3.18%	0.000	
	262144	63.0ns ± 3%	61.3ns ± 3%	-2.78%	0.000	

Table E.8: Performance of the *AlphaDev* C++ Sort3,4,5 implementations on type **uint64**. Performance is benchmarked using the official LLVM microbenchmark.

cpu	sort$K$	original LLVM sort	*AlphaDev*	% change	p-value	runs
Intel Skylake	1	3.97ns ± 5%	3.68ns ± 5%	-7.43%	0.000	
	3	5.28ns ± 7%	2.07ns ± 7%	-60.68%	0.000	
	4	5.88ns ± 7%	1.81ns ± 7%	-69.16%	0.000	
	5	7.22ns ± 8%	2.09ns ± 6%	-71.12%	0.000	
	16	11.0ns ± 8%	11.3ns ± 8%	+2.06%	0.000	
	64	19.8ns ±11%	19.8ns ± 10%	$\sim$	0.265	
	256	27.9ns ±8%	28.0ns ± 7%	+0.37%	0.044	
	1024	36.0ns ± 8%	36.2ns ± 7%	+0.46%	0.018	
	16384	52.1ns ± 6%	52.3ns ± 5%	+0.54%	0.001	
	262144	67.6ns ± 6%	68.4ns ± 7%	+1.22%	0.000	
ARMv8	1	4.01ns ± 0%	3.85ns ± 0%	-3.81%	0.000	
	3	4.79ns ± 0%	2.16ns ± 0%	-54.92%	0.000	
	4	5.55ns ± 0%	2.17ns ± 0%	-60.98%	0.000	
	5	6.69ns ± 0%	2.73ns ± 0%	-59.21%	0.000	
	16	10.6ns ± 0%	10.2ns ± 0%	-3.81%	0.000	
	64	18.1ns ± 0%	17.5ns ± 0%	-3.01%	0.000	
	256	25.1ns ± 0%	24.4ns ± 0%	-2.88%	0.000	
	1024	32.1ns ± 0%	31.3ns ± 0%	-2.61%	0.000	
	16384	45.8ns ± 1%	44.9ns ± 1%	-1.96%	0.000	
	262144	59.0ns ± 2%	58.1ns ± 2%	-1.55%	0.000	
AMD Zen 2	1	3.39ns ± 2%	3.39ns ± 0%	$\sim$	0.921	
	3	5.33ns ± 1%	1.75ns ± 1%	-67.12%	0.000	
	4	6.28ns ± 2%	1.55ns ± 1%	-75.35%	0.000	
	5	8.12ns ± 1%	1.74ns ± 1%	-78.60%	0.000	
	16	12.3ns ± 1%	11.1ns ± 1%	-9.73%	0.000	
	64	21.9ns ± 1%	21.0ns ± 1%	-4.21%	0.000	
	256	31.3ns ± 1%	30.5ns ± 1%	-2.75%	0.000	
	1024	40.8ns ± 1%	39.9ns ± 1%	-2.02%	0.000	
	16384	59.7ns ± 2%	59.0ns ± 1%	-1.31%	0.000	
	262144	78.1ns ± 3%	77.4ns ± 3%	-0.80%	0.000	

Table E.9: Performance of the *AlphaDev* C++ Sort3,4,5 implementations on type **float**. Performance is benchmarked using the official LLVM microbenchmark.

F Theorem: the Assembly Zero-One Principle

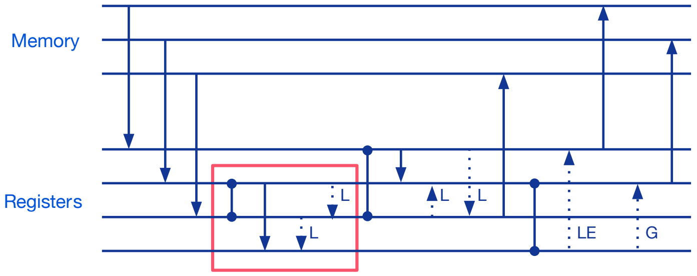

Figure F.4: An example Assembly Sorting Network for a Sort3 function. Note that three memory locations and four registers are required to generate a sort3 function. As a result, there are three wires that represent the memory locations and four wires to represent the register locations. A solid line arrow represents a move instruction from source to destination; a dotted line arrow is a conditional move where L, LE and G correspond to 'Less than', 'Less than or Equal to' and 'Greater than' respectively; a regular solid line represents a comparator from a classic sorting network.

In the sort setting, a computational bottleneck for *AlphaDev* lies in the reward computation, whose complexity grows super-exponentially as it requires executing the candidate program on all weak orderings of input sequences, whose counts are referred to as ordered Bell numbers [15]. For N inputs, the ordered Bell number is defined as $\texttt{ordBell}(N) = \sum_{k=1}^{N} \left\{ {N \atop k} \right\} k!$. For $N = 6$, the reward computation becomes costly enough to slow down the training considerably. For larger N, the problem becomes intractable. In this section, we optimize this correctness evaluation procedure by leveraging the Zero-One principle, and adapting it to assembly programs.

The Zero-One Principle.

Proposition 1. *[Zero-One Principle, 3] If a sorting network sorts all binary sequences in $\{0,1\}^N$, then it also sorts all sequences of arbitrary numbers correctly.*

This drastically reduces the number of sequences needed for evaluation. For example, sort8 only requires $2^8 = 256$ sequences instead of $8^8 = 16777216$. By adapting the zero-one principle to our setup, the number of input sequences required for testing correctness reduces dramatically as seen in Table F.10. We next go through the proof of the Assembly Zero-One principle, and start by introducing Assembly Sorting Networks.

F.1 Assembly Sorting Network

We define in this section a particular generalization of traditional sorting networks in the assembly language space that we coin as *"Assembly Sorting Networks"*. In this formulation, we have N memory wires and N_{reg} registers yielding in total $K = N + N_{\text{reg}}$ wires, and a special flag variable. An example is shown in Figure F.4. Each assembly instruction is mapped to a visual representation on the diagram. Formally,

Definition 1. *(Assembly Network) This network consists of N memory wires, N_{reg} register wires that carry values, one flag wire taking values in $\{$Less (L), Greater (G), Equal (E)$\}$, and a sequence of m operations $(Op^1, Op^2, \ldots, Op^m)$.*

Given an input sequence a, the state of an Assembly Sorting Network W after t operations is described by the wires-flag pair $(W^t[a], F^t[a])$, and we use the subscript notation $W_i^t[a]$ to denote the value at wire i. For simplicity, we sometimes omit the input sequence a in the notation when it is non-ambiguous.

Definition 2. *(Assembly Sorting Network) An Assembly Sorting Network is an Assembly Network designed to sort any input of length N by supporting the following set of operations:*

Definition 3. *(Move) The move operation mov<p,q> copies the value from wire p to wire q. At stage t, it produces the state (W^t, F^t) such that*

N	$2^N - 1$	$N!$	$\texttt{ordBell}(N)$	N^N
1	1	1	1	1
2	3	2	3	4
3	7	6	13	27
4	15	24	75	256
5	31	120	541	3125
6	63	720	4683	46656
7	127	5040	47293	823543
8	255	40320	545835	16777216

Table F.10: Sorting sequences needed for evaluating correctness. This table provides a comparison of the number of input sequences needed for the observation space, and evaluating correctness, as a function of the sort length N. We compare using the zero-one principle (2^N), total orderings without ties ($N!$), weak orderings ($\texttt{ordBell}(N)$) and total orderings (N^N)

- $W_i^t = \begin{cases} W_p^{t-1}, & \textit{if } i = q \\ W_i^{t-1}, & \textit{if } i \neq q \end{cases}$
- $F^t = F^{t-1}$

Definition 4. *(Compare) The compare operation cmp<p,q> compares the wires* p *and* q *and writes the result to the flag. At stage* t*, it produces the state* (W^t, F^t) *such that*

- $W^t = W^{t-1}$
- $F^t = \begin{cases} \textit{Less}, & \textit{if } W_p^{t-1} < W_q^{t-1} \\ \textit{Equal}, & \textit{if } W_p^{t-1} = W_q^{t-1} \\ \textit{Greater}, & \textit{if } W_p^{t-1} > W_q^{t-1} \end{cases}$

Definition 5. *(Conditional Move) The conditional move operation cmovLE<p,q> may move the value from wire* p *to* q *depending on the value of the flag, and thus of the result of the latest compare operation. At stage* t *produces the state* (W^t, F^t) *such that*

- $W_q^t = \begin{cases} W_p^{t-1} & \textit{if } F^{t-1} \in \{\textit{Less, Equal}\} \\ W_q^{t-1} & \textit{else} \end{cases}$
- $W_i^t = W_i^{t-1} \qquad \textit{if } i \neq q$
- $F^t = F^{t-1}$

The other conditional moves cmovL, cmovG, cmovGE, are defined similarly by replacing $\{$*Less, Equal*$\}$ *above with* $\{$*Less*$\}$, $\{$*Greater*$\}$, $\{$*Greater, Equal*$\}$ *respectively.*

Assumption 1. *We only allow a cmovXX<p,q> operation after there has been a cmp<r,s> operation so that the flag wire is initialized.*

F.2 Assembly Zero-One Principle

In this section, we study the conditions allowing to apply the zero-one principle described in Proposition 1 in the context of classic Sorting Networks to Assembly Sorting Networks.

Proposition 2. *The zero-one principle does not hold for Assembly Sorting Networks.*

Proof. We exhibit in Table 1 a counter-example program that sorts all binary sequences of length $N = 4$, but does not sort the integer sequence $(4, 3, 2, 1)$. □

As a counter-example to the zero-one principle for general Assembly Networks, we provide the following Program 1.

```
movl    (%rsi),   eax
movl    4(%rsi),  ecx
movl    8(%rsi),  edx
movl    12(%rsi), edi
movl    ecx,      r8d
movl    edx,      ebx
cmpl    edi,      r8d
cmovle  edi,      ecx
cmovgel edi,      r8d
cmpl    eax,      r8d
cmovle  eax,      edi
cmovgel eax,      r8d
cmpl    ecx,      ebx
cmovgel r8d,      ebx
cmovle  ecx,      edx
cmovgel ecx,      r8d
movl    ebx,      (%rsi)
cmpl    edi,      r8d
cmovgel edi,      ebx
movl    ebx,      4(%rsi)
cmpl    edx,      edi
cmovgel edx,      r8d
movl    r8d,      8(%rsi)
cmpl    eax,      edx
cmovle  eax,      edx
movl    edx,      12(%rsi)
```

Listing 1: Length-26 program which sorts binary inputs but not integer inputs.

As we show in the sequel, the reason of this negative result comes from the use of conditional moves, that can fail to preserve a monotony property in some situations. Consequently, in order to maintain the zero-one principle, we need to restrict ourselves to a smaller subclass of Assembly Sorting Networks, for which conditional moves are only allowed in certain places. Our next result characterizes these conditional moves.

Theorem F.1 (Assembly Zero-One Principle)**.** *An Assembly Sorting Network such that every conditional move operation is applied to values that fall within the range of the values of the previous compare operation, satisfies the zero-one principle.*

Formally, consider an Assembly Sorting Network W, let Op^t denote any conditional move operation cmovXX<p,q> in the network, applied at time t on wires p, q, and Op^k its latest preceding compare operation cmp<u,v>, where $k < t$. If all of the below four conditions are true,

(i) For every Op^t = cmovL<p,q>,

$$W_u^k < W_v^k \text{ implies } W_p^{t-1}, W_q^{t-1} \in [W_u^k, W_v^k]$$

(ii) For every Op^t = cmovLE<p,q>,

$$W_u^k > W_v^k \text{ implies } W_p^{t-1}, W_q^{t-1} \in [W_v^k, W_u^k]$$

(iii) For every Op^t = cmovG<p,q>,

$$W_u^k > W_v^k \text{ implies } W_p^{t-1}, W_q^{t-1} \in [W_v^k, W_u^k]$$

(iv) For every Op^t = *cmovGE<p,q>*,

$$W_u^k < W_v^k \textit{ implies } W_p^{t-1}, W_q^{t-1} \in [W_u^k, W_v^k],$$

then W *satisfies the zero-one principle.*

Proof. The full derivation can be seen in the Appendix F.3. ☐

This result allows us to exhibit a subclass of Assembly Sorting Networks for which the zero-one principle holds, which means that their correctness can be efficiently checked using binary sequences only. By making online decisions about which actions are legal, i.e. whether we should allow or not a cmovXX in the current state, we are guaranteed to stay in this subclass of verifiable networks. Unfortunately, the four conditions of Theorem F.1 are expressed in terms of arbitrary values flowing through the network, which means that checking them still requires evaluating this network on all possible input sequences $a \in [1, N]^N$, which defeats the initial purpose.

Consequently, to provide a practical implementation, we introduce another criterion that can be easily assessed on binary sequences only, and which we prove to be a sufficient condition for the four properties of Theorem F.1.

Theorem F.2. *(Practical implementation) For an Assembly Sorting Network* W*, consider an arbitrary* Op^t = *cmovXX<p,q> and the latest preceding* Op^k = *cmp<u,v>. If it holds that, for any* ***binary*** *input sequence* $b \in \{0, 1\}^N$

$$W_u^k[b] = W_v^k[b] \textit{ implies } W_p^{t-1}[b] = W_q^{t-1}[b] = W_u^k[b] \tag{1}$$

then W *verifies the four conditions of Theorem F.1, and thus the zero-one principle.*

Proof. The full derivation can be seen in the Appendix F.4. ☐

By restricting the search space, and despite the potential suboptimality that it could introduce, this result enables us to check the correctness of a sorting program by only testing the set of binary inputs, of size 2^N, rather than the set of integer inputs, of size $\texttt{ordBell}(N)$. This allows us to scale using a similar computational budget from $N = 6$ inputs up to $N = 8$.

F.3 Proof of Theorem F.1

Following Cormen et al. [16, Chapter 28.2], we will rely on the notion of monotonically increasing functions. We start by showing the following lemma.

Lemma F.1. *If an Assembly Sorting Network* W *commutes with any monotonically increasing function* f*, that is,*

$$W[f(a)] = f(W[a]), \quad \forall a \in [1, N]^N,$$

then it satisfies the zero-one principle of Proposition 1.

Proof. The proof follows the same steps as that of Cormen et al. [16, Theorem 28.2].

Let W be an Assembly Sorting Network that commutes with any monotonically increasing function f, we will show by contradiction that it satisfies the zero-one principle. Assume for the sake of contradiction that this principle does not hold, *i.e.* W sorts all binary sequences but there exists a sequence a of arbitrary numbers that the network does not correctly sort: a contains elements a_i and a_j such that $a_i < a_j$, but the network places a_j before a_i in the output sequence. We define the threshold function $f : x \to \mathbb{1}\{x \geq a_j\}$, which is monotonically increasing. Since the network commutes with f by assumption, and places a_j before a_i in the output sequence when a is input, it also places $f(a_j)$ before $f(a_i)$ in the output sequence when $f(a)$ is input. But since $f(a_i) = 0$ and $f(a_j) = 1$, we obtain the contradiction that the network fails to sort the binary sequence $f(a)$ correctly.

☐

For classic sorting networks, this commutative property was not needed because it was a direct consequence of sorting binary inputs. This is no longer the case with Assembly Sorting Networks, and we show next that it is now a consequence of the four conditions enumerated in Theorem F.1.

Proposition 3. *Consider an Assembly Sorting Network* W *that satisfies the conditions of Theorem F.1. Then,* W *commutes with any monotonically increasing function* f*:*

$$W[f(a)] = f(W[a]), \quad \forall a \in [1, N]^N \tag{2}$$

Proof. We prove this proposition by induction with respect to the number m of operations in the Assembly Network. We wish to show the following property.

Property $P(m)$**.** for any Assembly Network W with m operations,

$$W^m[f(a)] = f(W^m[a]), \quad \forall a \in [1, N]^N \tag{3}$$

Base case $P(0)$**.** The Assembly Network has no operations. Then W is the identity, $W^0[a] = a$, and the property holds.

Induction:. We assume the $P(k)$ holds for any $k < m$,

$$W^k[f(a)] = f(W^k[a]) \text{ for any Assembly Network } W \text{ with } k < m \text{ operations,}$$

and we wish to prove that $P(m)$ also holds.

Let W be an Assembly Network with m operations, $a \in [1, N]^N$ an input sequence, and f a monotonically increasing function. Let us consider the last operation of W: it can be one of cmp, mov, cmovXX. The following lemmas will deal with each instruction, showing that all of them maintain the commutativity of W^m with respect to monotonically increasing functions, which concludes the proof. □

Lemma F.2. *If Op*^{m} *= cmp<p,q>, then* (3) *holds.*

Proof. Since *cmp*<p,q> does not change the values on any wire, other than the flag wire, we have that $W^m = W^{m-1}$. Thus, applying the inductive hypothesis to W^{m-1} yields the desired result. □

Lemma F.3. *If Op*^{m} *= mov<p,q>, then* (3) *holds.*

Proof. For any wire $i \neq q$, *mov*<p,q> does not change the value of wire i, so again $W_i^m = W_i^{m-1}$ and we can conclude by applying the inductive property. On the other hand, the wire q is changed unconditionally and $W_q^m = W_p^{m-1}$. Thus, by the inductive hypothesis:

$$W_q^m[f(a)] = W_p^{m-1}[f(a)] = f(W_p^{m-1}[a]) = f(W_q^m[a])$$

□

Lemma F.4. *If Op*^{m} *= cmovL<p,q>, then* (3) *holds.*

Proof. We denote the preceding compare operation as Op^k = *cmp*<u,v> where $k < m$. As before, for all wires $i \neq q$, the values are unchanged, so the commutativity directly stems from the inductive hypothesis for these wires. Let us now consider the wire q.

By definition of *cmov*L<p,q>:

$$W_q^m = \begin{cases} W_p^{m-1} & \text{if } W_u^k < W_v^k \\ W_q^{m-1} & \text{if } W_u^k \geq W_v^k \end{cases}$$

Applying it to $f(a)$,

$$W_q^m[f(a)] = \begin{cases} W_p^{m-1}[f(a)] & \text{if } W_u^k[f(a)] < W_v^k[f(a)] \\ W_q^{m-1}[f(a)] & \text{if } W_u^k[f(a)] \geq W_v^k[f(a)] \end{cases}$$

By the inductive hypothesis applied to W^k, this can be rewritten as

$$W_q^m[f(a)] = \begin{cases} W_p^{m-1}[f(a)] & \text{if } f(W_u^k[a]) < f(W_v^k[a]) \\ W_q^{m-1}[f(a)] & \text{if } f(W_u^k[a]) \geq f(W_v^k[a]) \end{cases}$$

We consider two cases

Case 1: $W_u^k[a] \geq W_v^k[a]$**.** Since f is monotonically increasing, $f(W_u^k[a]) \geq f(W_v^k[a])$. Thus,

$$\begin{aligned} W_q^m[f(a)] &= W_q^{m-1}[f(a)] && \text{By definition of } cmov\text{L<p,q> applied to } f(a) \\ &= f(W_q^{m-1}[a]) && \text{By inductive hypothesis} \\ &= f(W_q^m[a]) && \text{By definition of } cmov\text{L<p,q> applied to } a \end{aligned}$$

Case 2: $W_u^k[a] < W_v^k[a]$**.** Since f is monotonically increasing, this implies $f(W_u^k[a]) \leq f(W_v^k[a])$.
If the inequality is strict, $f(W_u^k[a]) < f(W_v^k[a])$, then similarly to case 1:

$$W_q^m[f(a)] = W_p^{m-1}[f(a)] = f(W_p^{m-1}[a]) = f(W_q^m[a])$$

Conversely, if the inequality is an equality, $f(W_u^k[a]) = f(W_v^k[a])$, then

- f is constant on the interval $[W_u^k[a], W_v^k[a]]$, since it is monotonically increasing
- the two sequences a and $f(a)$ do not fall into the same case:

$$\text{we have } W_q^m[f(a)] = W_q^{m-1}[f(a)] \text{ while } W_q^m[a] = W_p^{m-1}[a]. \tag{4}$$

This is where the conditions of Theorem F.1 come in. By applying condition (i), we have that $W_p^{m-1}[a]$ and $W_q^{m-1}[a] \in [W_u^k[a], W_v^k[a]]$. Since f is constant in that interval, we can deduce that these two values are equal, which yields

$$\begin{aligned}
W_q^m[f(a)] &= W_q^{m-1}[f(a)] && \text{By (4)} \\
&= f(W_q^{m-1}[a]) && \text{By induction hypothesis} \\
&= f(W_p^{m-1}[a]) && \text{Since } f \text{ is constant on } [W_u^k[a], W_v^k[a]] \\
&= f(W_q^m[a]) && \text{By (4)}
\end{aligned}$$

□

Lemma F.5. *If Op^m = cmovLE<p,q>, cmovG<p,q> or cmovGE<p,q>, then* (3) *holds.*

Proof. The proofs for each operation is similar to that of Lemma F.4. It involves writing the definitions of $W_q^m[a]$ and $W_q^m[f(a)]$, and looking at their respective orders. When it is the same, they both do the conditional move from p to q, or none of them do, and the inductive hypothesis can be applied to obtain the result. The equality case $f(W_u^k[a]) = f(W_v^k[a])$ has to be treated separately, by noticing that f is then constant on that interval, which yields the equality of $W_p^{m-1}[a]$ and $W_q^{m-1}[a]$ by invoking the conditions (ii)–(iv), and enables to conclude the proof. □

F.4 Proof of Theorem F.2

Proof. We are going to show by induction the following property.

Property $P(m)$**.** Let W be an Assembly Sorting Network of length m which satisfies for any binary sequence $b \in \{0,1\}^N$

$$W_u^k[b] = W_v^k[b] \text{ implies } W_p^{t-1}[b] = W_q^{t-1}[b] = W_u^k[b] \tag{1}$$

where $t \leq m$ is the index of any Op$_t$ = *cmov*XX<p,q> and $k < t$ the index of the latest preceding Op$_k$ = *cmp*<u,v>.
Then, W also satisfies the four conditions of (i)–(iv) of Theorem F.1

Initialization $P(1)$**.** Any network of length 1 satisfies the conditions of Theorem F.1.

Induction $P(m-1) \implies P(m)$**.** We assume that $P(m-1)$ holds and prove $P(m)$.
Let W be an Assembly Sorting Network of length m which satisfies for any binary sequence $b \in \{0,1\}^N$

$$W_u^k[b] = W_v^k[b] \text{ implies } W_p^{t-1}[b] = W_q^{t-1}[b] = W_u^k[b]$$

We are going to show by contradiction that the four conditions (i)–(iv) of Theorem F.1 hold.

Consider the subprograms W^k for any $k \leq m-1$. They also satisfy the previous equality (1) for any $t \leq m-1$, so by the inductive hypothesis they satisfy the four conditions of Theorem F.1. In particular, by Proposition 3 they commute with any monotonically increasing function f:

$$W^k[f(a)] = f(W^k[a]), \quad \forall a, \forall k \leq m-1$$

Let t be the index of a $\mathrm{Op}_t =$ *cmov*XX<p,q> in W. If $t < m$, applying the inductive hypothesis to the subnetwork W^{m-1} directly yields the desired result that all four properties (i)–(iv) are satisfied, so the only case left to consider is when $t = m$: we are adding a conditional move at the end of the network. Let us consider the four conditions separately, depending on the type of conditional move, and prove each of them by contradiction

Case 1: Op^m = *cmov*L<p,q>. Assume that the condition (i) if false. Then, we have that $W_u^k < W_v^k$ but one of W_p^{m-1} or W_q^{m-1} does not belong to $[W_u^k, W_v^k]$.

- **Example case:** $W_u^k < W_v^k < W_p^{m-1}$
  Define the monotonically increasing threshold function $f : x \to \mathbb{1}\{x \geq W_p^{m-1}\}$, and for any input sequence a consider its binarization $b = f(a)$. Then, we have

$$\begin{aligned}
W_u^k[b] &= W_u^k[f(a)] \\
&= f(W_u^k[a]) && \text{Since } W^k \text{ commutes with } f \\
&= 0 && \text{By definition of } f \\
&= f(W_v^k[a]) && \text{By definition of } f \\
&= W_v^k[f(a)] && \text{Since } W^k \text{ commutes with } f \\
&= W_v^k[b]
\end{aligned}$$

  Since we assumed (1), this implies that $W_p^{m-1}[b] = W_q^{m-1}[b] = W_u^k[b]$, that is, $W_p^{m-1}[f(a)] = W_q^{m-1}[f(a)] = W_u^k[f(a)]$. Since W^{m-1} commutes with f, this implies the contradiction $1 = f(W_p^{m-1}[a]) = f(W_u^k[a]) = 0$. Thus, the condition (i) must be true.

- **Other cases**: We can proceed similarly : whenever one of the values W_p^{m-1} or W_q^{m-1} is outside $[W_u^k, W_v^k]$, simply define f as a threshold function that separates this value from the interval, and applying W to $f(a)$ will lead to the same contradiction in (1).

Cases 2–4: Op^m = *cmov*LE<p,q>, *cmov*G<p,q> or *cmov*GE<p,q>. Again, we can proceed similarly to prove the conditions (ii)–(iv), respectively. Each time, we have to show that W_p^{m-1} and W_q^{m-1} belong to an interval, and we can proceed by contradiction as in Case 1, by defining a threshold function f that separates the interval from the value that is outside of it. Applying this function on the input sequence f will yield a contradiction in (1).
All conditions (i)–(iv) hold, so $P(m)$ is true, which concludes the induction.

□

G Additional programs

In this section, we provide a list of 12 correct sort3, sort4 and sort5 assembly programs discovered by *AlphaDev*. In the list, the naming convention is sort**X_Y**, where X is the number of elements to be sorted and Y is the number of assembly instructions in this program. As can be seen by the programs, *AlphaDev* is capable of finding multiple optimal solutions (e.g., a variant of the sort3 17 instruction program - Sort3_17). In addition, while *AlphaDev* is trying to find the optimal sorting program for a given length, it discovers less efficient, but correct programs such as sort3_18 and sort3_19. In this case, these longer programs have one and two extra *mov*<A,B> instructions respectively.

- Sort3_17
- Sort3_18
- Sort3_19
- Sort3_20
- Sort4_28
- Sort4_29

- Sort4_30
- Sort4_31
- Sort5_42
- Sort5_43
- Sort5_44
- Sort5_45

```
/*Copyright 2022 DeepMind Technologies Limited.  Licensed under the Apache License,
↪  Version 2.0 and CC BY 4.0. You may not use this file except in compliance with these
↪  licenses. Copies of the licenses can be found at
↪  https://www.apache.org/licenses/LICENSE-2.0 and
↪  https://creativecommons.org/licenses/by/4.0/legalcode.*/

#include <algorithm>
#include <functional>
#include <utility>
#include <vector>

#include <gtest/gtest.h>

void Sort3_17(int* buffer) {
  asm volatile(
      "mov 0x4(%0), %%r8d             \n"
      "mov 0x8(%0), %%edx             \n"
      "mov 0x8(%0), %%ecx             \n"
      "cmp %%r8d, %%ecx               \n"
      "cmovge %%r8d, %%edx            \n"
      "cmovl %%r8d, %%ecx             \n"
      "mov %%ecx, %%r8d               \n"
      "mov (%0), %%eax                \n"
      "cmp %%eax, %%r8d               \n"
      "cmovge %%eax, %%r8d            \n"
      "cmovl %%eax, %%ecx             \n"
      "mov %%ecx, 0x8(%0)             \n"
      "cmp %%edx, %%eax               \n"
      "cmovle %%edx, %%r8d            \n"
      "cmovg %%edx, %%eax             \n"
      "mov %%r8d, 0x4(%0)             \n"
      "mov %%eax, (%0)                \n"
      : "+r"(buffer)
      :
      : "eax", "ecx", "edx", "r8d", "memory");
}

void Sort3_18(int* buffer) {
  asm volatile(
      "mov (%0), %%r8d                \n"
      "mov 0x8(%0), %%edx             \n"
      "mov %%edx, %%ecx               \n"
      "mov 0x4(%0), %%eax             \n"
```

```
      "cmp %%eax, %%edx              \n"
      "cmovg %%eax, %%edx            \n"
      "cmovl %%eax, %%ecx            \n"
      "cmp %%r8d, %%ecx              \n"
      "cmovle %%ecx, %%r8d           \n"
      "mov (%0), %%eax               \n"
      "cmovle %%eax, %%ecx           \n"
      "cmp %%eax, %%edx              \n"
      "cmovle %%edx, %%eax           \n"
      "mov %%ecx, 0x8(%0)            \n"
      "cmovle %%r8d, %%edx           \n"
      "mov %%edx, 0x4(%0)            \n"
      "mov %%edx, %%ecx              \n"
      "mov %%eax, (%0)               \n"
      : "+r"(buffer)
      :
      : "eax", "ecx", "edx", "r8d", "memory");
}

void Sort3_19(int* buffer) {
  asm volatile(
      "mov 0x8(%0), %%ecx            \n"
      "mov (%0), %%r8d               \n"
      "mov 0x4(%0), %%edx            \n"
      "cmp %%edx, %%ecx              \n"
      "cmovle %%ecx, %%edx           \n"
      "mov 0x8(%0), %%eax            \n"
      "mov 0x4(%0), %%eax            \n"
      "cmovl %%eax, %%ecx            \n"
      "cmp %%r8d, %%ecx              \n"
      "cmovle %%ecx, %%r8d           \n"
      "mov (%0), %%eax               \n"
      "cmovle %%eax, %%ecx           \n"
      "cmp %%eax, %%edx              \n"
      "cmovle %%edx, %%eax           \n"
      "cmovge %%edx, %%r8d           \n"
      "mov %%eax, (%0)               \n"
      "mov %%r8d, 0x4(%0)            \n"
      "mov 0x4(%0), %%edx            \n"
      "mov %%ecx, 0x8(%0)            \n"
      : "+r"(buffer)
      :
      : "eax", "ecx", "edx", "r8d", "memory");
}

void Sort3_20(int* buffer) {
  asm volatile(
      "mov (%0), %%eax               \n"
      "mov 0x4(%0), %%eax            \n"
      "mov 0x8(%0), %%ecx            \n"
      "mov %%eax, %%edx              \n"
      "cmp %%eax, %%ecx              \n"
      "cmovl %%ecx, %%edx            \n"
      "cmovl %%eax, %%ecx            \n"
```

```
      "mov (%0), %%eax                \n"
      "mov %%eax, %%r8d               \n"
      "cmp %%r8d, %%ecx               \n"
      "cmovle %%ecx, %%r8d            \n"
      "cmovl %%eax, %%ecx             \n"
      "cmp %%edx, %%eax               \n"
      "cmp %%eax, %%edx               \n"
      "cmovl %%edx, %%eax             \n"
      "cmovg %%edx, %%r8d             \n"
      "cmp %%ecx, %%eax               \n"
      "mov %%ecx, 0x8(%0)             \n"
      "mov %%r8d, 0x4(%0)             \n"
      "mov %%eax, (%0)                \n"
      : "+r"(buffer)
      :
      : "eax", "ecx", "edx", "r8d", "memory");
}


void Sort4_28(int* buffer) {
  asm volatile(
      "mov (%0), %%eax                \n"
      "mov 0x4(%0), %%ecx             \n"
      "mov 0x8(%0), %%edx             \n"
      "cmp %%eax, %%edx               \n"
      "mov %%ecx, %%r9d               \n"
      "mov (%0), %%r8d                \n"
      "cmovg %%edx, %%r8d             \n"
      "mov %%r8d, %%r10d              \n"
      "cmovg %%eax, %%edx             \n"
      "mov 0xc(%0), %%eax             \n"
      "cmp %%ecx, %%eax               \n"
      "cmovge %%eax, %%r9d            \n"
      "cmovge %%ecx, %%eax            \n"
      "cmp %%r10d, %%r9d              \n"
      "cmovge %%r9d, %%r10d           \n"
      "cmovge %%r8d, %%r9d            \n"
      "mov %%r10d, 0xc(%0)            \n"
      "mov %%edx, %%r10d              \n"
      "cmp %%edx, %%eax               \n"
      "cmovge %%eax, %%r10d           \n"
      "cmovge %%edx, %%eax            \n"
      "mov %%eax, (%0)                \n"
      "cmp %%r9d, %%r10d              \n"
      "mov %%r9d, %%edx               \n"
      "cmovg %%r10d, %%edx            \n"
      "mov %%edx, 0x8(%0)             \n"
      "cmovge %%r9d, %%r10d           \n"
      "mov %%r10d, 0x4(%0)            \n"
      : "+r"(buffer)
      :
      : "eax", "ecx", "edx", "r8d", "r9d", "memory");
}
```

```
void Sort4_29(int* buffer) {
  asm volatile(
      "mov (%0), %%eax                \n"
      "mov 0x4(%0), %%ecx             \n"
      "mov %%ecx, %%r9d               \n"
      "mov 0x8(%0), %%edx             \n"
      "mov %%eax, %%r8d               \n"
      "cmp %%r8d, %%edx               \n"
      "cmovg %%edx, %%r8d             \n"
      "cmovge %%eax, %%edx            \n"
      "mov 0xc(%0), %%eax             \n"
      "cmp %%ecx, %%eax               \n"
      "cmovge %%eax, %%r9d            \n"
      "cmovge %%ecx, %%eax            \n"
      "cmp %%r8d, %%r9d               \n"
      "mov %%r8d, %%r10d              \n"
      "cmovge %%r9d, %%r10d           \n"
      "cmovg %%r8d, %%r9d             \n"
      "cmp %%edx, %%eax               \n"
      "mov %%r10d, 0xc(%0)            \n"
      "mov %%r9d, 0x8(%0)             \n"
      "mov %%edx, %%r10d              \n"
      "cmovg %%eax, %%r10d            \n"
      "cmovge %%edx, %%eax            \n"
      "mov %%r9d, %%edx               \n"
      "cmp %%r9d, %%r10d              \n"
      "mov %%eax, (%0)                \n"
      "cmovg %%r10d, %%edx            \n"
      "mov %%edx, 0x8(%0)             \n"
      "cmovge %%r9d, %%r10d           \n"
      "mov %%r10d, 0x4(%0)            \n"
      : "+r"(buffer)
      :
      : "eax", "ecx", "edx", "r8d", "r9d", "memory");
}

void Sort4_30(int* buffer) {
  asm volatile(
      "mov 0x4(%0), %%ecx             \n"
      "mov 0x8(%0), %%edx             \n"
      "mov (%0), %%eax                \n"
      "cmp %%eax, %%edx               \n"
      "mov %%eax, %%r8d               \n"
      "mov (%0), %%eax                \n"
      "cmovg %%edx, %%r8d             \n"
      "cmovge %%eax, %%edx            \n"
      "mov 0xc(%0), %%eax             \n"
      "cmp %%ecx, %%eax               \n"
      "mov %%ecx, %%r9d               \n"
      "cmovg %%eax, %%r9d             \n"
      "cmovge %%eax, %%r9d            \n"
      "cmovg %%ecx, %%eax             \n"
      "cmp %%r8d, %%r9d               \n"
      "mov %%r8d, %%r10d              \n"
```

```
      "cmovge %%r9d, %%r10d          \n"
      "mov %%r10d, 0xc(%0)           \n"
      "cmovg %%r8d, %%r9d            \n"
      "mov %%edx, %%r10d             \n"
      "cmp %%edx, %%eax              \n"
      "cmovge %%eax, %%r10d          \n"
      "cmovge %%edx, %%eax           \n"
      "mov %%eax, (%0)               \n"
      "cmp %%r9d, %%r10d             \n"
      "mov %%r9d, %%edx              \n"
      "cmovge %%r10d, %%edx          \n"
      "mov %%edx, 0x8(%0)            \n"
      "cmovge %%r9d, %%r10d          \n"
      "mov %%r10d, 0x4(%0)           \n"
      : "+r"(buffer)
      :
      : "eax", "ecx", "edx", "r8d", "r9d", "memory");
}

void Sort4_31(int* buffer) {
  asm volatile(
      "mov (%0), %%eax               \n"
      "mov 0x4(%0), %%ecx            \n"
      "mov 0x8(%0), %%edx            \n"
      "mov (%0), %%eax               \n"
      "cmp %%eax, %%edx              \n"
      "mov %%eax, %%r8d              \n"
      "cmovg %%edx, %%r8d            \n"
      "cmovge %%eax, %%edx           \n"
      "cmp %%ecx, %%eax              \n"
      "mov 0xc(%0), %%eax            \n"
      "cmp %%ecx, %%eax              \n"
      "mov %%ecx, %%r9d              \n"
      "cmovg %%eax, %%r9d            \n"
      "cmovge %%eax, %%r9d           \n"
      "cmovg %%ecx, %%eax            \n"
      "cmp %%r8d, %%r9d              \n"
      "mov %%r8d, %%r10d             \n"
      "cmovge %%r9d, %%r10d          \n"
      "mov %%r10d, 0xc(%0)           \n"
      "cmovg %%r8d, %%r9d            \n"
      "mov %%edx, %%r10d             \n"
      "cmp %%edx, %%eax              \n"
      "cmovge %%eax, %%r10d          \n"
      "cmovge %%edx, %%eax           \n"
      "mov %%eax, (%0)               \n"
      "cmp %%r9d, %%r10d             \n"
      "mov %%r9d, %%edx              \n"
      "cmovge %%r10d, %%edx          \n"
      "mov %%edx, 0x8(%0)            \n"
      "cmovg %%r9d, %%r10d           \n"
      "mov %%r10d, 0x4(%0)           \n"
      : "+r"(buffer)
      :
```

```
      : "eax", "ecx", "edx", "r8d", "r9d", "memory");
}

void Sort5_42(int* buffer) {
  asm volatile(
      "mov (%0), %%eax                \n"
      "mov %%eax, %%ecx               \n"
      "mov 0x4(%0), %%edx             \n"
      "cmp %%edx, %%eax               \n"
      "cmovg %%edx, %%eax             \n"
      "cmovl %%edx, %%ecx             \n"
      "mov 0x8(%0), %%r8d             \n"
      "mov 0xc(%0), %%r9d             \n"
      "mov %%r8d, %%r10d              \n"
      "cmp %%r9d, %%r10d              \n"
      "cmovl %%r9d, %%r10d            \n"
      "mov 0x10(%0), %%edx            \n"
      "cmovge %%r9d, %%r8d            \n"
      "mov %%r8d, %%r9d               \n"
      "cmp %%eax, %%r9d               \n"
      "cmovge %%eax, %%r8d            \n"
      "cmovge %%r9d, %%eax            \n"
      "cmp %%edx, %%r10d              \n"
      "mov %%edx, %%r9d               \n"
      "cmovg %%r10d, %%edx            \n"
      "cmovg %%r9d, %%r10d            \n"
      "cmp %%r9d, %%r8d               \n"
      "cmovle %%r8d, %%r9d            \n"
      "cmovle %%r10d, %%r8d           \n"
      "mov %%r9d, (%0)                \n"
      "cmp %%ecx, %%r10d              \n"
      "cmovge %%ecx, %%r8d            \n"
      "cmovl %%ecx, %%r10d            \n"
      "cmp %%eax, %%r8d               \n"
      "mov %%eax, %%r9d               \n"
      "cmovge %%r8d, %%eax            \n"
      "cmovg %%r9d, %%r8d             \n"
      "mov %%r8d, 0x4(%0)             \n"
      "cmp %%r10d, %%edx              \n"
      "cmovle %%edx, %%r10d           \n"
      "cmovge %%edx, %%ecx            \n"
      "mov %%ecx, 0x10(%0)            \n"
      "cmp %%eax, %%r10d              \n"
      "cmovl %%r10d, %%eax            \n"
      "cmovge %%r10d, %%r9d           \n"
      "mov %%eax, 0x8(%0)             \n"
      "mov %%r9d, 0xc(%0)             \n"
      : "+r"(buffer)
      :
      : "eax", "ecx", "edx", "r8d", "r9d", "r10d", "memory");
}

void Sort5_43(int* buffer) {
  asm volatile(
```

```
      "mov (%0), %%eax               \n"
      "mov 0x4(%0), %%ecx            \n"
      "cmp %%eax, %%ecx              \n"
      "mov %%eax, %%edx              \n"
      "cmovl %%ecx, %%edx            \n"
      "cmovg %%ecx, %%eax            \n"
      "mov 0xc(%0), %%r8d            \n"
      "mov 0x10(%0), %%ecx           \n"
      "cmp %%r8d, %%ecx              \n"
      "mov %%r8d, %%r9d              \n"
      "cmovl %%ecx, %%r9d            \n"
      "cmovg %%ecx, %%r8d            \n"
      "mov 0x8(%0), %%r10d           \n"
      "cmp %%r10d, %%r8d             \n"
      "mov %%r10d, %%ecx             \n"
      "cmovl %%r8d, %%ecx            \n"
      "cmovle %%r10d, %%r8d          \n"
      "cmp %%ecx, %%r9d              \n"
      "cmovle %%r9d, %%r10d          \n"
      "cmovg %%r9d, %%ecx            \n"
      "cmp %%eax, %%r8d              \n"
      "mov %%eax, %%r9d              \n"
      "cmovl %%r8d, %%r9d            \n"
      "cmovle %%eax, %%r8d           \n"
      "cmp %%edx, %%ecx              \n"
      "mov %%edx, %%eax              \n"
      "cmovl %%ecx, %%eax            \n"
      "cmovle %%edx, %%ecx           \n"
      "mov %%r8d, 0x10(%0)           \n"
      "cmp %%eax, %%r10d             \n"
      "cmovle %%r10d, %%edx          \n"
      "mov %%edx, (%0)               \n"
      "cmovg %%r10d, %%eax           \n"
      "cmp %%r9d, %%ecx              \n"
      "mov %%r9d, %%r8d              \n"
      "cmovl %%ecx, %%r8d            \n"
      "cmovle %%r9d, %%ecx           \n"
      "mov %%ecx, 0xc(%0)            \n"
      "cmp %%r8d, %%eax              \n"
      "cmovle %%eax, %%r9d           \n"
      "mov %%r9d, 0x4(%0)            \n"
      "cmovg %%eax, %%r8d            \n"
      "mov %%r8d, 0x8(%0)            \n"
      : "+r"(buffer)
      :
      : "eax", "ecx", "edx", "r8d", "r9d", "r10d", "memory");
}

void Sort5_44(int* buffer) {
  asm volatile(
      "mov (%0), %%eax               \n"
      "mov 0x4(%0), %%ecx            \n"
      "mov 0xc(%0), %%r9d            \n"
      "mov 0x10(%0), %%r10d          \n"
```

```
      "mov %%eax, %%r8d              \n"
      "mov 0x8(%0), %%edx            \n"
      "cmp %%eax, %%ecx              \n"
      "cmovle %%ecx, %%r8d           \n"
      "cmovle %%eax, %%ecx           \n"
      "mov 0xc(%0), %%eax            \n"
      "cmp %%r9d, %%r10d             \n"
      "cmovg %%r10d, %%r9d           \n"
      "cmovle %%r10d, %%eax          \n"
      "cmovle %%r9d, %%r10d          \n"
      "cmp %%edx, %%r10d             \n"
      "cmovg %%edx, %%r9d            \n"
      "cmovl %%edx, %%r10d           \n"
      "cmp %%eax, %%edx              \n"
      "cmovle %%eax, %%r9d           \n"
      "cmovge %%eax, %%edx           \n"
      "mov %%edx, 0x4(%0)            \n"
      "mov %%ecx, %%eax              \n"
      "cmp %%ecx, %%r10d             \n"
      "cmovge %%r10d, %%ecx          \n"
      "cmovle %%r10d, %%eax          \n"
      "mov %%ecx, 0x10(%0)           \n"
      "mov %%r8d, %%ecx              \n"
      "cmp %%ecx, %%r9d              \n"
      "cmovle %%r9d, %%ecx           \n"
      "mov %%eax, %%r10d             \n"
      "cmovl %%r8d, %%r9d            \n"
      "cmp %%edx, %%r8d              \n"
      "cmovge %%edx, %%r8d           \n"
      "cmovle %%edx, %%ecx           \n"
      "cmp %%eax, %%r9d              \n"
      "cmovl %%r9d, %%r10d           \n"
      "mov %%r8d, (%0)               \n"
      "cmovl %%eax, %%r9d            \n"
      "cmp %%eax, %%edx              \n"
      "cmovl %%ecx, %%eax            \n"
      "cmovg %%ecx, %%r10d           \n"
      "mov %%eax, 0x4(%0)            \n"
      "mov %%r10d, 0x8(%0)           \n"
      "mov %%r9d, 0xc(%0)            \n"
      : "+r"(buffer)
      :
      : "eax", "ecx", "edx", "r8d", "r9d", "r10d", "memory");
}

void Sort5_45(int* buffer) {
  asm volatile(
      "mov (%0), %%eax               \n"
      "mov 0x4(%0), %%r8d            \n"
      "mov 0xc(%0), %%r9d            \n"
      "mov %%r8d, %%ecx              \n"
      "mov 0x10(%0), %%r10d          \n"
      "mov 0x8(%0), %%edx            \n"
      "cmp %%eax, %%r8d              \n"
```

```
        "cmovg %%eax, %%r8d            \n"
        "cmovle %%eax, %%ecx           \n"
        "mov %%ecx, 0x4(%0)            \n"
        "cmp %%r9d, %%r10d             \n"
        "mov %%r8d, (%0)               \n"
        "mov %%r9d, %%eax              \n"
        "cmovle %%r10d, %%eax          \n"
        "cmovl %%r9d, %%r10d           \n"
        "cmovge %%r10d, %%r9d          \n"
        "cmp %%edx, %%r9d              \n"
        "cmovle %%edx, %%r10d          \n"
        "cmovg %%edx, %%r9d            \n"
        "cmp %%eax, %%edx              \n"
        "cmovle %%eax, %%r9d           \n"
        "cmovg %%eax, %%edx            \n"
        "cmp %%ecx, %%r10d             \n"
        "mov %%r10d, %%eax             \n"
        "cmovge %%ecx, %%r10d          \n"
        "cmovl %%ecx, %%eax            \n"
        "mov %%r9d, %%ecx              \n"
        "cmp %%r8d, %%r9d              \n"
        "mov %%eax, 0x10(%0)           \n"
        "cmovl %%r8d, %%r9d            \n"
        "cmovg %%r8d, %%ecx            \n"
        "mov %%r10d, %%eax             \n"
        "cmp %%eax, %%r9d              \n"
        "cmovle %%r9d, %%r10d          \n"
        "cmovle %%eax, %%r9d           \n"
        "cmp %%r8d, %%edx              \n"
        "mov %%r9d, 0xc(%0)            \n"
        "cmovl %%edx, %%r8d            \n"
        "cmovge %%edx, %%ecx           \n"
        "mov %%r8d, (%0)               \n"
        "cmp %%eax, %%ecx              \n"
        "cmovge %%ecx, %%r10d          \n"
        "mov %%r10d, 0x8(%0)           \n"
        "cmovl %%ecx, %%eax            \n"
        "mov %%eax, 0x4(%0)            \n"
        : "+r"(buffer)
        :
        : "eax", "ecx", "edx", "r8d", "r9d", "r10d", "memory");
}
```

References

[1] Julian Schrittwieser et al. “Mastering Atari, Go, Chess and Shogi by Planning with a Learned Model”. In: *Nature* 588.7839 (2020), pp. 604–609.

[2] Marc G Bellemare, Will Dabney, and Rémi Munos. “A distributional perspective on reinforcement learning”. In: *International Conference on Machine Learning*. PMLR. 2017, pp. 449–458.

[3] Sanguthevar Rajasekaran and Sandeep Sen. “A generalization of the 0–1 Principle for sorting”. In: *Information processing letters* 94.1 (2005), pp. 43–47.

[4] Toshio Nakatani et al. “K-way bitonic sort”. In: *IEEE Transactions on Computers* 38.2 (1989), pp. 283–288.

[5] Peter W Battaglia et al. “Relational inductive biases, deep learning, and graph networks”. In: *arXiv preprint arXiv:1806.01261* (2018).

[6] Julian Schrittwieser et al. “Mastering atari, go, chess and shogi by planning with a learned model”. In: *Nature* 588.7839 (2020), pp. 604–609.

[7] Julian Schrittwieser et al. “Online and offline reinforcement learning by planning with a learned model”. In: *arXiv preprint arXiv:2104.06294* (2021).

[8] Chris Cummins et al. “Programl: Graph-based deep learning for program optimization and analysis”. In: *arXiv preprint arXiv:2003.10536* (2020).

[9] Miltiadis Allamanis, Marc Brockschmidt, and Mahmoud Khademi. “Learning to Represent Programs with Graphs”. In: *International Conference on Learning Representations*. 2018.

[10] Marco Gori, Gabriele Monfardini, and Franco Scarselli. “A new model for learning in graph domains”. In: *Proceedings. 2005 IEEE International Joint Conference on Neural Networks, 2005.* Vol. 2. IEEE. 2005, pp. 729–734.

[11] Franco Scarselli et al. “The graph neural network model”. In: *IEEE transactions on neural networks* 20.1 (2008), pp. 61–80.

[12] Google. *VarInt Protocol Buffer Serialization and Deserialization*. Version 0.2.5. 2022. URL: https://developers.google.com/protocol-buffers/docs/encoding.

[13] AtCoder. *A Buttons*. 2022. URL: https://atcoder.jp/contests/abc124/tasks/abc124_a (visited on 01/07/2022).

[14] Yujia Li et al. “Competition-level code generation with alphacode”. In: *Science* (2022).

[15] Wikipedia. *Ordered Bell Numbers*. https://en.wikipedia.org/wiki/Ordered_Bell_number. 2021.

[16] Thomas H Cormen et al. *Introduction to algorithms*. MIT press, 2009.
